# Exosomal PGE2 from M2 macrophages inhibits neutrophil recruitment and NET formation through lipid mediator class switching in sepsis

**DOI:** 10.1186/s12929-023-00957-9

**Published:** 2023-08-02

**Authors:** Yang Jiao, Ti Zhang, Mei Liu, Luyang Zhou, Mengzhi Qi, Xin Xie, Xueyin Shi, Xiaoping Gu, Zhengliang Ma

**Affiliations:** 1 Department of Anesthesiology, Nanjing Drum Tower Hospital, The Affliated Hospital of Nanjing University Medical School, 321 Zhongshan Road, Nanjing, 210008 China; 2 Department of Anesthesiology and Intensive Care Unit, Xinhua Hospital, School of Medicine, Shanghai Jiaotong University, 1665 Kongjiang Road, Shanghai, 200092 China; 3 National Clinical Research Center of Kidney Diseases, Jinling Hospital, Nanjing University School of Medicine, Nanjing, China; 4 Department of Intensive Care Unit, Nanjing Drum Tower Hospital, The Affliated Hospital of Nanjing University Medical School, Nanjing, China

**Keywords:** M2 macrophage-derived exosomes, Neutrophils, Migration, Neutrophil extracellular traps, Lipid mediator class switching

## Abstract

**Background:**

Excess polymorphonuclear neutrophil (PMN) recruitment or excessive neutrophil extracellular trap (NET) formation can lead to the development of multiple organ dysfunction during sepsis. M2 macrophage-derived exosomes (M2-Exos) have exhibited anti-inflammatory activities in some inflammatory diseases to mediate organ functional protection, but their role in treating sepsis-related acute lung injury (ALI) remains unclear. In this study, we sought to investigate whether M2-Exos could prevent potentially deleterious inflammatory effects during sepsis-related ALI by modulating abnormal PMN behaviours.

**Methods:**

C57BL/6 wild-type mice were subjected to a caecal ligation and puncture (CLP) mouse model to mimic sepsis in vivo*,* and M2-Exos were administered intraperitoneally 1 h after CLP. H&E staining, immunofluorescence and immunohistochemistry were conducted to investigate lung tissue injury, PMN infiltration and NET formation in the lung. We further demonstrated the role of M2-Exos on PMN function and explored the potential mechanisms through an in vitro coculture experiment using PMNs isolated from both healthy volunteers and septic patients.

**Results:**

Here, we report that M2-Exos inhibited PMN migration and NET formation, alleviated lung injury and reduced mortality in a sepsis mouse model. In vitro, M2-Exos significantly decreased PMN migration and NET formation capacity, leading to lipid mediator class switching from proinflammatory leukotriene B4 (LTB4) to anti-inflammatory lipoxin A4 (LXA4) by upregulating 15-lipoxygenase (15-LO) expression in PMNs. Treatment with LXA4 receptor antagonist attenuated the effect of M2-Exos on PMNs and lung injury. Mechanistically, prostaglandin E2 (PGE2) enriched in M2-Exos was necessary to increase 15-LO expression in PMNs by functioning on the EP4 receptor, upregulate LXA4 production to downregulate chemokine (C-X-C motif) receptor 2 (CXCR2) and reactive oxygen species (ROS) expressions, and finally inhibit PMN function.

**Conclusions:**

Our findings reveal a previously unknown role of M2-Exos in regulating PMN migration and NET formation through lipid mediator class switching, thus highlighting the potential application of M2-Exos in controlling PMN-mediated tissue injury in patients with sepsis.

**Supplementary Information:**

The online version contains supplementary material available at 10.1186/s12929-023-00957-9.

## Introduction

Sepsis is a prevalent disease worldwide and one of the leading causes of hospital death, characterized by infection-triggered immune hyperactivation and cytokine storms, which cause tissue damage and eventually lead to multiple organ dysfunction syndrome (MODS) [1]. Acute respiratory distress syndrome (ARDS) is the most common severe manifestation of MODS and an important factor contributing to the morbidity and mortality of sepsis [2].

Polymorphonuclear neutrophils (PMNs), the most abundant leukocytes in mammals, reach the inflammatory site in a cascade-like manner, where they activate specific effector functions such as the release of reactive oxygen species (ROS), degranulation, formation of neutrophil extracellular traps (NETs), and phagocytosis [3]. After fulfilling the appropriate effector functions, dampening PMN activation and infiltration is crucial to prevent damage to the host [4]. However, the aberrant recruitment or activation of PMNs is one of the hallmarks of ARDS [5]. Therefore, modulation of PMN recruitment and function during sepsis to justify the beneficial antimicrobial function and the potentially deleterious inflammatory effect has gained increasing interest over the years.

Macrophages are also known as key mediators in determining the outcome of inflammatory responses. Macrophages are divided into two phenotypically distinct populations: proinflammatory and anti-inflammatory/proresolving macrophages, named M1 and M2 macrophages, respectively. In general, M2 macrophages appear at later stages of infection to control and resolve inflammation and repair tissues [6]. The crosstalk between PMNs and macrophages in regulating inflammation during sepsis-related ARDS has been documented [7]. M2 macrophages function to clear PMNs and remove NETs via phagocytosis accumulated in inflammatory sites [8].

Exosomes, an important form of extracellular vesicle, have been demonstrated to mediate intercellular communication in various physical processes [9]. Recently, M2 macrophage-derived exosomes (M2-Exos) have been revealed to play an immunoprotective role in inflammatory disease and mediating organ functional protection [10, 11]. We and others have also demonstrated that exosomes are novel mediators between PMNs and macrophages during sepsis [7, 12]. However, the roles of M2 macrophage-derived exosomes in PMN recruitment and function during sepsis remain unclear.

In addition, previous studies have demonstrated that prostaglandin E2 (PGE2) produced by local macrophages switches lipid mediator biosynthesis from predominantly proinflammatory leukotriene B4 [LTB4; 5-lipoxygenase (5-LO)-initiated pathway] to anti-inflammatory lipoxin A4 (LXA4), a 15-LO product in PMNs [13, 14]. LTB4 initiates and amplifies PMN chemotaxis and NET formation, and these effects can be reversed by LXA4, which is considered an endogenous “stop signal” in inflammation [15]. Switching from LTB4 to LXA4 production marks the resolution phase [16]. However, whether intrinsic lipid mediator class switching controls neutrophil phenotype alteration in sepsis needs to be further addressed.

In this study, we first identified that M2-Exos could alleviate lung injury and reduce mortality by inhibiting PMN migration and NET formation during sepsis. A mechanistic study revealed that M2-Exo-PGE2 switched lipid mediator biosynthesis from LTB4 to LXA4 in PMNs by increasing 15-LO expression. These findings suggest a previously unidentified role of M2-Exos in sepsis and may help us better understand the endogenous mechanisms for the resolution of inflammation and lead to the development of novel therapeutic approaches.

## Materials and methods

### Patient samples and ethics statement

EDTA-anticoagulated venous blood (20 mL) was collected from patients with an early (less than 24 h) diagnosis of sepsis who were admitted to the ICU of Nanjing Drum Tower Hospital (Nanjing, China) between January and November 2022. The Third International Consensus Definitions for Sepsis and Septic Shock (Sepsis-3) were used to diagnose sepsis. Pregnant women and patients under 18 years of age and those with severe anaemia, active bleeding, or chemotherapy were excluded. The demographic data of the included septic patients are shown in Additional file 1: Table S1. Blood samples from healthy volunteers were used as controls, and healthy blood donors were age- and sex-matched to septic patients. Then, plasma and neutrophils were isolated from healthy donors and septic patients. All plasma samples were aliquoted and stored at – 80 °C. The study was conducted in accordance with the Declaration of Helsinki, and the protocol was approved by the institutional ethics and review board of Nanjing Drum Tower Hospital (Approval No. 2022-257-01). Informed consent was obtained from the participating volunteers, patients or their representatives.

### Macrophage cell culture

Human peripheral blood was obtained from healthy volunteers with informed consent. Peripheral blood mononuclear cells (PBMCs) were isolated using a Ficoll 1.077 density gradient (Solarbio, Beijing, China) as described previously [17]. PBMCs were differentiated into macrophages by incubation with Rosewell Park Memorial Institute (RPMI) 1640 medium (Gibco, USA) containing 10% fetal bovine serum (FBS, Gibco, USA) and 20 ng/mL recombinant human macrophage-colony stimulating factor (rhM-CSF, PeproTech, Suzhou, China). Mature M0 macrophages were induced after 7 days of culture. M2 macrophages were induced by adding 20 ng/mL IL-4 (PeproTech, China) for an additional 48 h. To inhibit PGE2 expression, celecoxib (20 µM; #HY-14398, MCE corporation, USA), a selective COX-2 inhibitor, was added to the culture medium.

In addition, we also polarized mouse Raw264.7 macrophages to M2-type macrophages by adding 20 ng/mL mouse IL-4 and IL-13 (PeproTech, China) to complete culture medium (DMEM containing 10% FBS, supplemented with 50 mg/mL penicillin/streptomycin) for 48 h. Then, RT‒qPCR was performed to identify the M2 polarization of the macrophages. To inhibit PGE2 expression, celecoxib (20 µM) was also added to the culture medium.

### Exosome isolation and characterization

After the induction of M0/M2 macrophages, the culture medium was replaced with RPMI 1640 containing 10% exosome-free FBS (#EXO-FBS-50A-1; System Biosciences, Palo Alto, CA, USA) for 24 h. Then, exosomes were isolated from the supernatant of M0 macrophages (M0-Exos) and M2 macrophages (M2-Exos) using ExoQuick-TC (#EXOTC10A-1; System Biosciences, USA) according to the manufacturer’s instructions. The detailed methods used to determine exosomal morphology, size distribution and surface marker expression are described in Additional file 2.

### PMN isolation

PMNs were isolated from the venous blood of healthy volunteers or septic patients by polymorphprep™ isolation reagent (#1114742, Axis-Shield, Norway) according to the manufacturer’s instructions. The resulting cells consisted of 90% PMNs, and the viability of the isolated PMNs was 95%, as assessed by flow cytometry and Trypan blue staining. After isolation, PMNs were suspended in complete culture medium (RPMI 1640 containing 1% FBS, supplemented with 50 mg/mL penicillin/streptomycin) at a concentration of 10^6^ cells/mL.

### In vitro co-culture experiments

Ex vivo neutrophil function analysis was performed on ICU admission samples using septic plasma. PMNs were activated upon adding 20% septic plasma (SP) sterilized through a 0.22-μm filter (Millipore) to the culture medium, and 20% plasma from healthy volunteers (HP) was used as a negative control. To rule out the possibility that plasma from different septic patients could influence the results, we treated PMNs isolated from the same individual using septic plasma from the same patient for each experiment, and every experiment was repeated at least three times using septic plasma from different patients. After the 1-h pretreatment with septic plasma, the supernatant was changed to fresh complete culture medium, and PMNs were then cocultured with PBS/M0-Exos/M2-Exos (100 μg/mL) derived from PBMC-differentiated macrophages for 5 h at 37 °C. For migration capacity analysis of neutrophils from septic patients, PMNs were directly cocultured with M0/M2-Exos (100 μg/mL) for 5 h after isolation.

In addition, some PMNs were cultured with or without 10 µM BOC-2 (LXA4 receptor antagonist; #HY-P1795, MCE corporation, USA), 1 µM PD146176 (15-LO inhibitor; #HY-103157, MCE corporation, USA), 1 µM E7046 (EP4 receptor antagonist; #HY-103088, MCE corporation, USA), 100 nM LXA4 (#90410, Cayman Chemical, Michigan, USA), and 100 nM PGE2 (#HY-101952, MCE corporation, USA).

### Transwell assay

After treatment with M0/M2-Exos for 5 h, neutrophils (2 × 10^5^) were collected and plated in the upper insert (polycarbonate filter 5 µm) of the Transwell kits (#3415, Corning, USA). Medium containing 20 ng/mL recombinant human IL-8 (Sino Biological, Beijing, China) was placed in the lower well as a chemotactic stimulus. After a 2-h incubation, cells in the lower chamber were counted under a microscope.

### NET quantification assay

To quantify NETs in the cell culture supernatant and plasma, we used the PicoGreen dsDNA Quantification Kit (Invitrogen, Carlsbad, CA, USA) and a capture ELISA based on myeloperoxidase (MPO) associated with DNA [18]. For ELISA analysis of NET concentration in plasma, 1 μg/mL anti-MPO mAb was used as a capture antibody with Cell Death Detection ELISA (Roche, Indianapolis, IN, USA) according to the instructions.

NET formation was also quantified by confocal microscopy. PMNs were allowed to settle on glass coverslips precoated with poly-l-lysine (#354085, Corning, NY, USA) for 30 min prior to being treated for a specific period of time. PMNs were incubated with 1 μM SYTOX Green reagent (#S7020, Invitrogen, USA) at 37 °C for 10 min. Nuclei were counterstained using DAPI, and the cells were mounted in Antifade Mounting Medium (#P0126, Beyotime Biotechnology, Shanghai, China) for imaging with a confocal microscope. For each slice, 5 random fields were captured and analysed. NET-positive cells and NET area were quantified using ImageJ software v.1.3.7. Only structures depicting NET morphology and positive for SYTOX Green were selected for area quantification, and intact granulocyte nuclei were excluded from the analysis.

### Targeted metabolite analysis of arachidonic acid (AA)-derived eicosanoids

Fourteen AA-derived eicosanoids in M0/M2-Exo-treated PMNs isolated from heathy volunteers and septic patients were detected by liquid chromatography-tandem mass spectrometry (LC‒MS/MS) according to the established method [19]. Approximately 50 mg of PMN pellets were collected and homogenized with methanol. After centrifugation (12,000 g, 4 °C, 15 min), the supernatant was collected for extraction of AA-derived eicosanoids with the Oasis HLB elution system (Waters, Milford, MA), which was preactivated and equilibrated with methanol and water. After elution with methanol, the eluted AA-derived eicosanoids were lyophilized and reconstituted in 1% formic acid and 80% acetonitrile solution for analysis with liquid chromatography high resolution mass spectrometry (Vanquish, UPLC coupled with Q Exactive, Thermo Fisher, USA). Electrospray ionization was used for detection in both positive and negative ion modes. The standards TXB2 (thromboxane B2), PGE2, PGD2 (prostaglandin D2), PGA2 (prostaglandin A2), PGJ2 (prostaglandin J2), 6-trans-LTB4 (6-trans leukotriene B4), LTB4, 15-HETE (15-hydroxyeicosatetraenoic acid), 12-HETE (12-hydroxyeicosatetraenoic acid), 5-HETE (5-hydroxyeicosatetraenoic acid), 14,15-EET (14,15-epoxy-5,8,11-eicosatrienoic acid), 5-oxo-ETE (5-oxo-6,8,11,14-eicosatetraenoic acid), 11,12-EET (11,12-epoxy-5,8,14-eicosatrienoic acid), and 8,9-EET (8,9-epoxyeicosatrienoic acid) were purchased from TIANGEN (Beijing, China). Quantitative control samples were used to monitor the stability and repeatability of the system. MultiQuant software was used to extract chromatographic peak area and retention time. Metabolite concentration was calculated based on the peak area ratios analyte/internal standard and concentration of standard samples.

### Animals

Wild-type (WT) male C57BL/6J mice aged 6–8 weeks (Vital River Laboratories, Zhejiang, China) were fed under a specific pathogen-free environment in the Laboratory Animal Center of Nanjing Drum Tower Hospital. All animal experiments were conducted under the rules approved by the Ethics Committee of Nanjing Drum Tower Hospital (Approval No. 2021AE01055).

### Establishment of the mouse model of caecal ligation and puncture (CLP) and in vivo exosome administration

The CLP mouse model was prepared as previously described [7]. Mice were sedated with an intraperitoneal injection of pentobarbital (60 mg/kg). After disinfection, a 1 cm midline laparotomy was made in the abdomen. The caecum was then exteriorized, ligated below the caecal valve, and punctured with an 18-gauge needle to induce sepsis. A small drop of caecal content was extruded. The caecum was then returned to the peritoneal cavity, and the abdominal incision was closed with sutures. The animals were resuscitated with (5 mL/100 g) saline. Sham animals underwent the same surgical procedures without caecum ligation and puncture. To explore exosome function in vivo, mice were intraperitoneally (*i.p.*) injected with M0/M2-Exos (300 μg/mouse) derived from mouse Raw264.7 macrophages using 31-gauge insulin syringes 1 h after CLP surgery. After 24 h of CLP, blood and lung tissues were harvested as described previously.

### Histopathological evaluation of lung tissues

To visualize changes in morphology, lung tissues were harvested and fixed in 4% paraformaldehyde for H&E staining. H&E staining was evaluated by a pathologist who was blinded to the experimental groups. To evaluate lung injury, five independent random lung fields were evaluated per mouse for neutrophils in alveolar spaces, neutrophils in interstitial spaces, hyaline membranes, proteinaceous debris filling the airspaces, and alveolar septal thickening. These results were weighed according to the official American Thoracic Society workshop report on features and measurements of experimental acute lung injury (ALI) in animals. The resulting injury score is a continuous value between 0 and 1.

### Bacterial load quantification

To determine the bacterial load, lungs were harvested at 24 h after CLP under sterile conditions. The same amounts of the lung tissues were homogenized, incubated at 37 °C for 1 h, and centrifuged at 500×*g* for 5 min. The supernatant of the tissues was then properly diluted with sterile normal saline and plated on tryptic soy agars. The agar plates were incubated at 37 °C for 24 h, and the number of bacterial colonies was calculated as colony forming units (CFU).

### Immunohistochemistry (IHC) and neutrophil quantification in the lung tissues

IHC was performed by treating the paraffin-embedded tissue sections with 3% H_2_O_2_ for 15 min and then subjecting them to microwave Ag retrieval. The slides were blocked with 3% BSA for 30 min and incubated with rabbit anti-mouse anti Ly-6G Ab overnight at 4 °C. After three washes with PBS, the samples were first incubated with HRP-conjugated goat anti-rabbit antibody and then with diaminobenzidine (Servicebio, Wuhan, China) as a substrate and counterstained with haematoxylin. Images were captured using an Olympus microscope (Olympus, Tokyo, Japan). Five fields per slide were selected randomly from a single mouse and then evaluated by an experienced pathologist blinded to the assignment of mice to treatments.

### Immunofluorescence (IF) staining

Paraffin-embedded lung tissues were sectioned, blocked with PBS containing 1% goat serum and 3% BSA, and permeabilized with PBS/0.01% Triton X-100. For neutrophil quantification, slides were incubated overnight at 4 °C with rabbit anti-mouse anti Ly-6G antibody. For NET detection in the lung tissue, the slides were incubated with antibodies against MPO and citrullinated histone H3 (CitH3) (#ab5103, Abcam). Following incubation with the primary antibody, the slides were washed with PBS, incubated for 1 h with species-specific secondary antibody coupled with Alexa Fluor Dye, counterstained with DAPI, and finally viewed under an Olympus microscope.

### Systemic circulating neutrophils

Systemic circulating neutrophils in mouse venous blood were detected using a BC-6800Plus instrument (Mindray, Shenzhen, China).

### Flow cytometry analysis of neutrophils

Single-cell suspensions were obtained from peripheral blood, labelled with fluorescent antibodies, and filtered through 40-μm cell strainers. All samples were analysed on a C6 instrument (BD Biosciences) and analysed with FlowJo software. The antibodies used for staining cells were as follows: FITC anti-mouse CD11b (#101205, Biolegend, USA), PE anti-mouse Gr-1 (#108407, Biolegend, USA), and APC anti-mouse CXCR2 (#149311, Biolegend, USA). Mouse neutrophils were defined as cells expressing both Gr-1 and CD11b.

### Detection of ROS

ROS generation in PMNs was determined by flow cytometry with CM-H2DCFDA staining (#C6827, Invitrogen, USA). Briefly, PMNs were collected and resuspended in prewarmed loading buffer containing freshly prepared probe (10 μM). After a 30-min incubation at room temperature, the fluorescence intensity was examined by flow cytometry.

### Enzyme-linked immunosorbent assay (ELISA)

The levels of LXA4, LTB4 and PGE2 were measured by ELISA kits (CUSABIO, Houston, USA) according to the manufacturer's protocols. The absorbance was measured at 450 nm with a microplate reader, and the levels of LXA4, LTB4 and PGE2 were calculated by the optical density and standard curve.

### Statistical analysis

Normally distributed data were tested using the Shapiro‒Wilk test and are presented as the means ± standard deviations. Comparisons between two groups were performed by the 2-tailed Student’s t test. Multiple group comparisons were performed by one-way ANOVA followed by Tukey’s multiple comparisons test with GraphPad Prism 8 software. Comparison of survival rates between groups was performed using the log-rank test. A value of *P* < 0.05 was considered statistically significant.

## Results

### M2-Exos reduce lung injury and improve the survival of septic mice

**Fig. 1:**
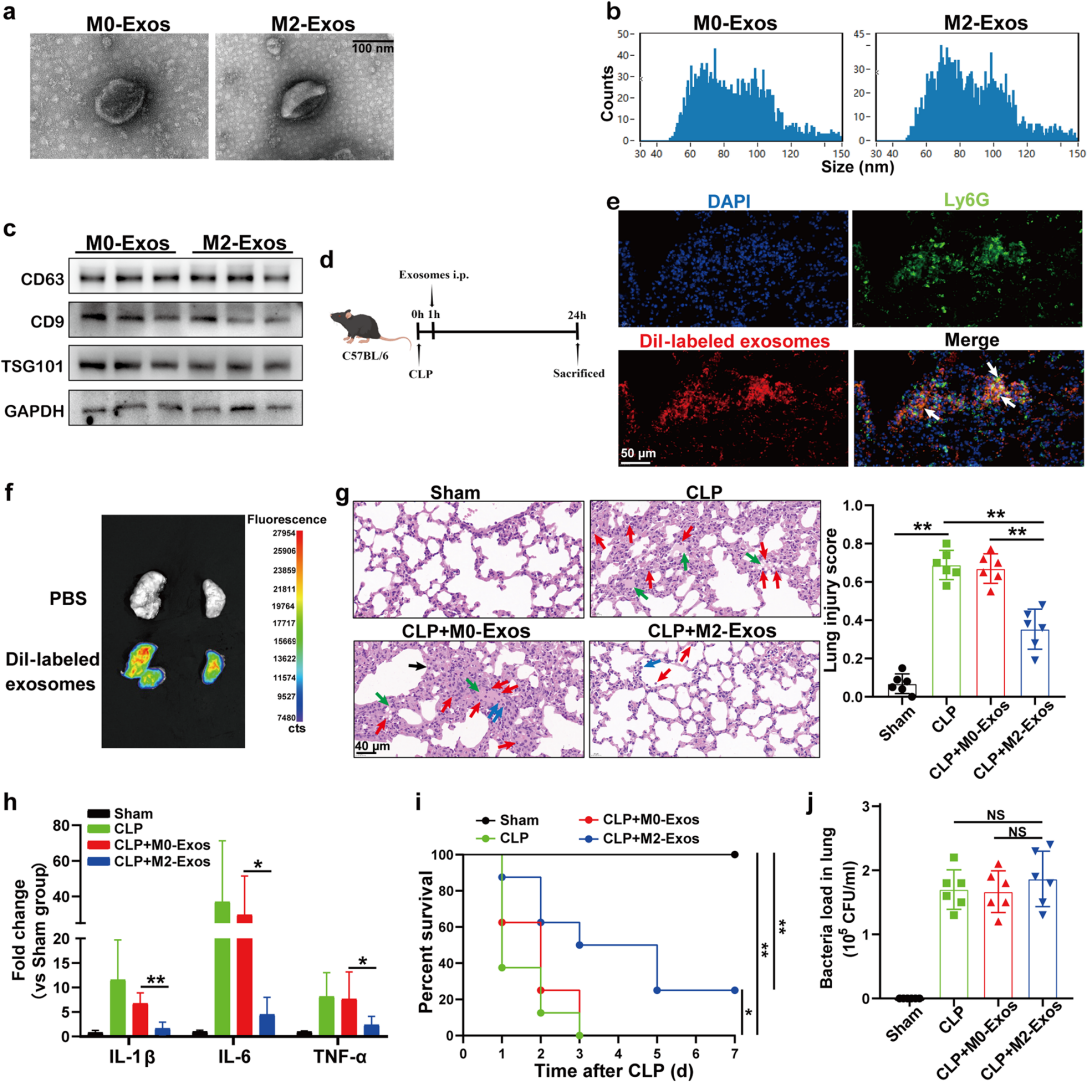
M2-Exos reduce lung injury and improve the survival of septic mice. **a** Electron micrograph of exosomes derived from the supernatant of polarized mouse Raw264.7 macrophages. Scale bar, 100 nm. **b** Exosome size distribution was measured by NanoSight tracking analysis. **c** CD63, CD9 and TSG101 protein expression in exosomes loaded with equal amounts of exosome protein (40 μg) was quantified by Western blotting. **d**–**j** WT C57BL/6 mice were intraperitoneally administered M0-Exos or M2-Exos (300 μg/mouse) derived from mouse Raw264.7 macrophages 1 h after CLP. **d** Schematic design for M0-Exo/M2-Exo treatment in the CLP model. **e** Representative images of direct immunofluorescence staining of DNA (blue), Ly6G (green), and Dil (red) in lung sections following intraperitoneal injection of Dil-labelled exosomes; white arrows indicate Dil-positive neutrophils. Scale bar, 50 μm. **f** Ex vivo fluorescent signals in the lungs of mice intraperitoneally injected with Dil-labelled exosomes. **g** Evaluation of lung histology by H&E staining (magnification × 400). Red arrows indicate neutrophils in the alveolar and interstitial space, blue arrows indicate alveolar macrophages, green arrows indicate proteinaceous debris filling, and black arrows indicate thickening of the alveolar walls. Scale bar, 40 μm. Lung injury scores were assessed. **h** Detection of inflammatory cytokine mRNA (IL-1β, IL-6, TNF-α) expression in lung tissues by RT‒qPCR. **i** Survival rate of CLP mice with M0-Exo or M2-Exo treatment (n = 8) and log-rank test was used for the analysis. **j** Lung tissues were harvested at 24 h after CLP. The supernatant was collected after homogenization and centrifugation. An equal amount of the supernatant was spread on agar plates for colony formation. The number of bacterial colonies was assessed. CFU, colony-forming unit. One-way analysis of variance with Tukey’s multiple comparisons test was used for the analysis. Graphs represent means ± standard deviations, n ≥ 3; *P < 0.05, **P < 0.01 compared within two groups; NS, not significant

First, exosomes were isolated and purified from the supernatant of mouse Raw264.7 macrophages that were cultured to the M0 phenotype (M0-Exos) or M2 phenotype (M2-Exos) and were characterized morphologically using transmission electron microscopy. As shown in Fig. 1a, the isolated microvesicles displayed a round, cup-shaped morphology and were approximately 100 nm in diameter. Furthermore, NanoSight analysis determined that the particle size distribution of the purified exosomes was approximately 100 nm (Fig. 1b), and western blotting showed high expression of the exosome-specific markers CD63, CD9 and TSG101 in M0-Exos and M2-Exos (Fig. 1c).

To investigate the function of M2-Exos in the biology of sepsis-related ALI/ARDS, we established a caecal ligation and puncture (CLP)-induced sepsis model that was clinically consistent with human septic peritonitis, and either M0-Exos or M2-Exos were administered intraperitoneally (*i.p.*) 1 h after surgery (Fig. 1d). Ex vivo fluorescence imaging showed that Dil-labelled exosomes accumulated in the lung tissue 24 h after intraperitoneal injection and colocalized with Ly6G (neutrophil-specific marker) (Fig. 1e, f). The histopathological appearance of the lung tissue showed marked accumulation of neutrophils and alveolar septal thickening following CLP surgery, and lung injury was attenuated after M2-Exo treatment (Fig. 1g). Consistent with the histological evaluation, intraperitoneal injection of M2-Exos significantly inhibited the expression of proinflammatory mediators (IL-6, IL-1β and TNF-α) in the lung tissue following CLP (Fig. 1h). Furthermore, compared with the M0-Exo-treated group, M2-Exo (150 or 300 µg/mouse) treatment significantly increased the survival of the animals (Fig. 1i and Additional file 1: Fig. S1), while the bacterial load in the lungs was not significantly different between the two groups (Fig. 1j). To determine whether M2-Exos affected CLP-induced tissue damage and mouse lethality at different stages, M0/M2-Exos (300 µg/mouse) were administered 1 day after CLP, and M2-Exos still showed a significant protective effect on lung injury and increased the survival rate of septic mice (Additional file 1: Fig. S2). All these data indicate that M2-Exos could alleviate lung injury and improve the survival rate of septic mice.

### M2-Exos inhibit PMN recruitment and NET formation during sepsis in vivo

**Fig. 2:**
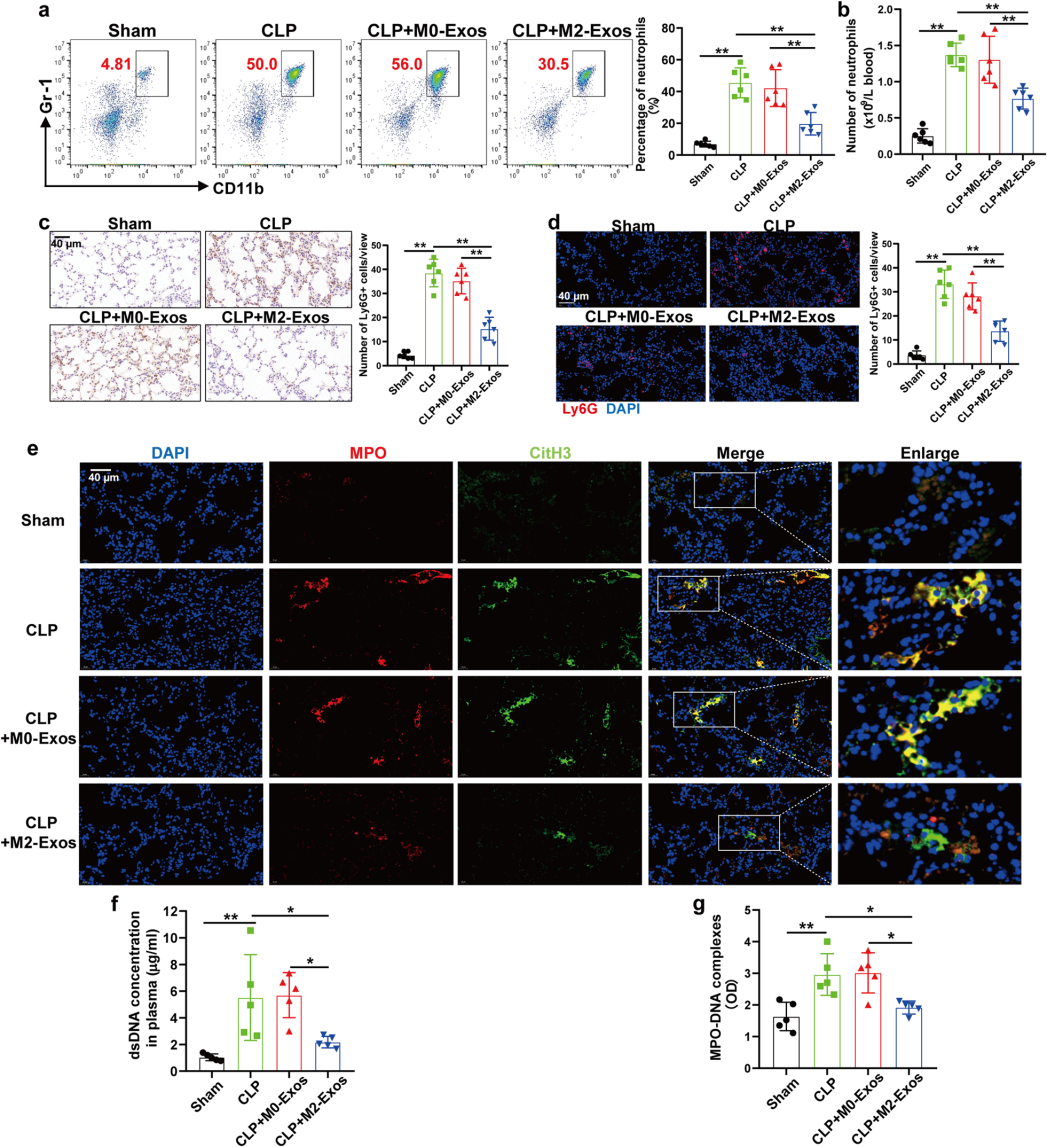
M2-Exos inhibit PMN recruitment and NET formation during sepsis in vivo. WT C57BL/6 mice were administered M0-Exos or M2-Exos (300 μg/mouse) derived from mouse Raw264.7 macrophages via intraperitoneal injection 1 h after surgery. Twenty-four hours after CLP, venous blood and lung tissues were harvested. **a** Flow cytometry detection of the percentage of systemic circulating PMNs by staining with CD11b and Gr-1. **b** Absolute neutrophil number in peripheral blood. **c** and **d** Ly6G^+^ cells in the lung tissues were detected by immunohistochemistry and immunofluorescence. Scale bar, 40 μm. **e** Representative images showing the presence of NETs (MPO, red; citrullinated H3, green) in the lung tissues. Nuclei were counterstained with DAPI (blue). Scale bar, 40 μm. **f** and **g** Quantification of dsDNA and circulating NET structures in the plasma of mice using PicoGreen fluorescent dye and MPO-DNA-ELISA, respectively. One-way analysis of variance with Tukey’s multiple comparisons test was used for the analysis. Graphs represent means ± standard deviations, n = 5–6; *P < 0.05, **P < 0.01 compared within two groups

The high lethality of sepsis is associated with dysregulation of the host inflammatory response, including the detrimental effect of aberrant activation of PMNs [20, 21]. Therefore, we decided to test whether M2-Exos could inhibit the overactivation of PMNs in sepsis. Intraperitoneal injection of M2-Exos significantly reduced the number of PMNs in the peripheral blood (Fig. 2a, b) and lung tissues (Fig. 2c, d) following CLP, indicating impaired recruitment of PMNs from the bone marrow to the circulation and eventually to the organ.

In addition, a previous study demonstrated that NETs were primarily observed in the lungs, and NETs facilitate bacterial clearance but can also directly induce cytotoxic effects on tissues, which in turn propagate inflammation and cause organ injury during sepsis [22]. In our study, immunofluorescence staining with anti-citrullinated histone H3 (CitH3) and anti-MPO showed that M2-Exo treatment reduced NET formation in the lung tissues of CLP mice (Fig. 2e). The amounts of plasma dsDNA and soluble NET components (MPO-DNA complexes) were significantly lower in M2-Exo-treated mice than in M0-Exo-treated controls (Fig. 2f, g).

### M2-Exos inhibit PMN recruitment and NET formation during sepsis in vitro

**Fig. 3:**
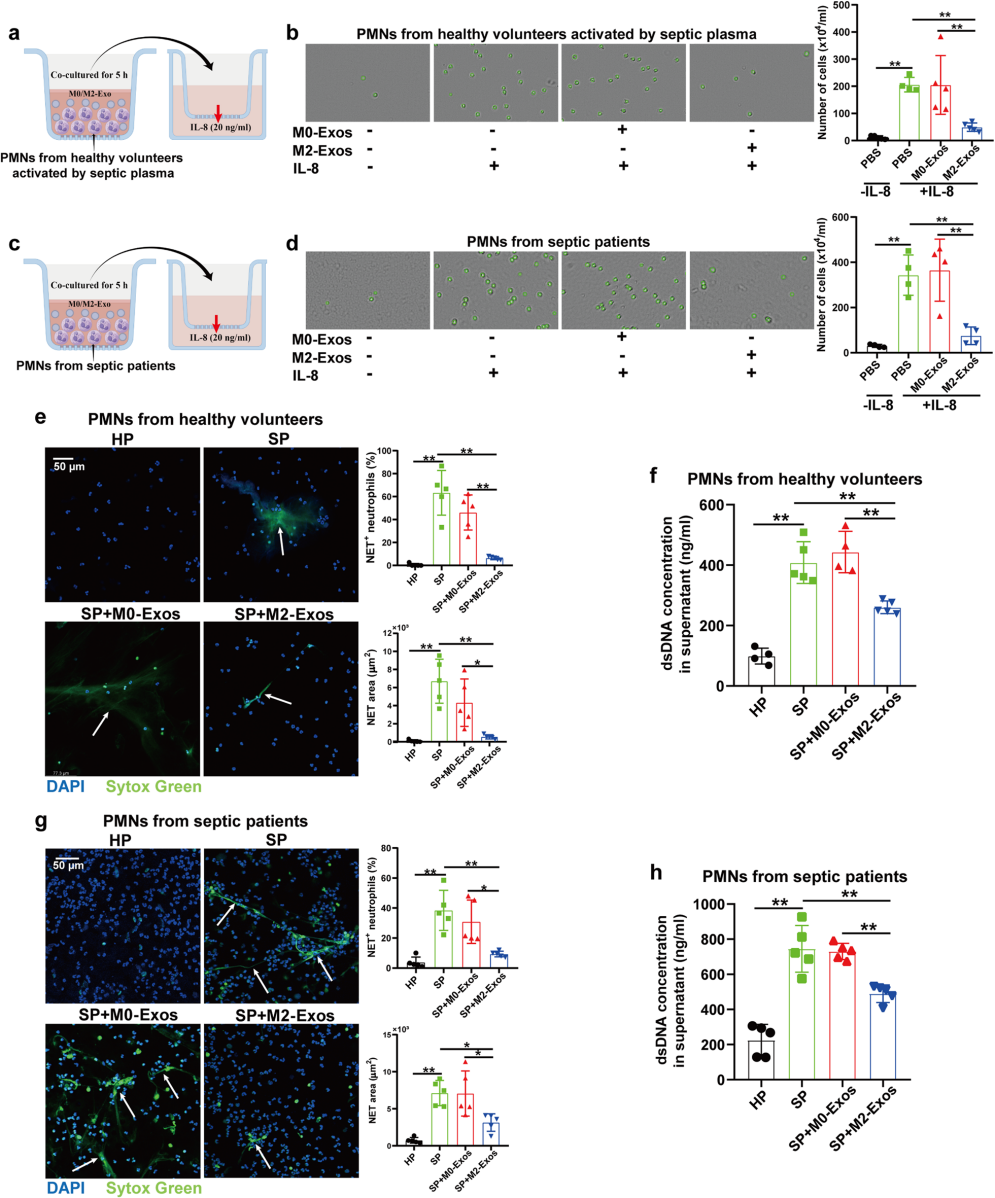
M2-Exos inhibit PMN recruitment and NET formation during sepsis in vitro. **a** and **b** PMNs from healthy volunteers were preactivated by septic plasma and then cocultured with M0/M2-Exos (100 μg/mL) derived from PBMC-differentiated macrophages. After 5 h, PMNs were collected for migration capacity analysis with IL-8 as a chemokine. After a 2-h incubation, cells in the lower chamber were collected and counted under a microscope. **c** and **d** PMNs isolated from septic patients were directly cocultured with M0/M2-Exos (100 μg/mL) derived from PBMC-differentiated macrophages for 5 h after isolation. Then, PMNs were transferred for the transwell assay. **e**–**h** Ex vivo NET formation assay with neutrophils isolated from healthy volunteers or septic patients activated by septic plasma (SP). Plasma from healthy volunteers (HP) was used as a negative control. Typical images of NET formation are presented in **e** and **g** using SYTOX Green (green), where white arrows indicate NETs. Scale bar, 50 μm. NET formation was quantified as the percentage of neutrophils forming NETs and the NET area per microscopic field. **f** and **h** Quantification of dsDNA in the supernatant of cultured PMNs using PicoGreen fluorescent dye. One-way analysis of variance with Tukey’s multiple comparisons test was used for the analysis. Graphs represent means ± standard deviations; *P < 0.05, **P < 0.01 compared within two groups

To further determine whether M2-Exos could limit the chemotaxis and NET formation capacity of PMNs, we established an in vitro coculture system. As illustrated in Fig. 3a, PMNs from healthy volunteers were first activated by septic plasma and then cocultured with 50–150 µg/mL M0/M2-Exos derived from human PBMC-differentiated macrophages. The characterization of exosomes and the phagocytosis of Dil-labelled exosomes by PMNs are shown in Additional file 1: Fig. S3. PMN migration towards IL-8 was decreased significantly after M2-Exo treatment compared with that in the M0-Exo-treated group (Fig. 3b, Additional file 1: Fig. S4a). In addition, the recruitment of PMNs isolated from septic patients towards IL-8 was also inhibited by M2-Exos (Fig. 3c, d, Additional file 1: Fig. S4b).

Subsequently, we observed typical NET structure formation in PMNs isolated from both healthy volunteers and septic patients after incubation with plasma from septic patients (SP) but not with plasma from healthy controls (HP). Upon culturing PMNs with M2-Exos, we found that NET formation induced by septic plasma was significantly reduced compared with that in the M0-Exo group (Fig. 3e–h). In addition, septic plasma could inhibit PMN apoptosis, while treatment with M2-Exos following septic plasma showed no effect on the apoptotic rate of PMNs (Additional file 1: Fig. S5). All these data suggest that M2-Exos possess the ability to inhibit PMN chemotaxis and NET production during sepsis.

### M2-Exos lead to lipid mediator class switching of PMNs during sepsis

**Fig. 4:**
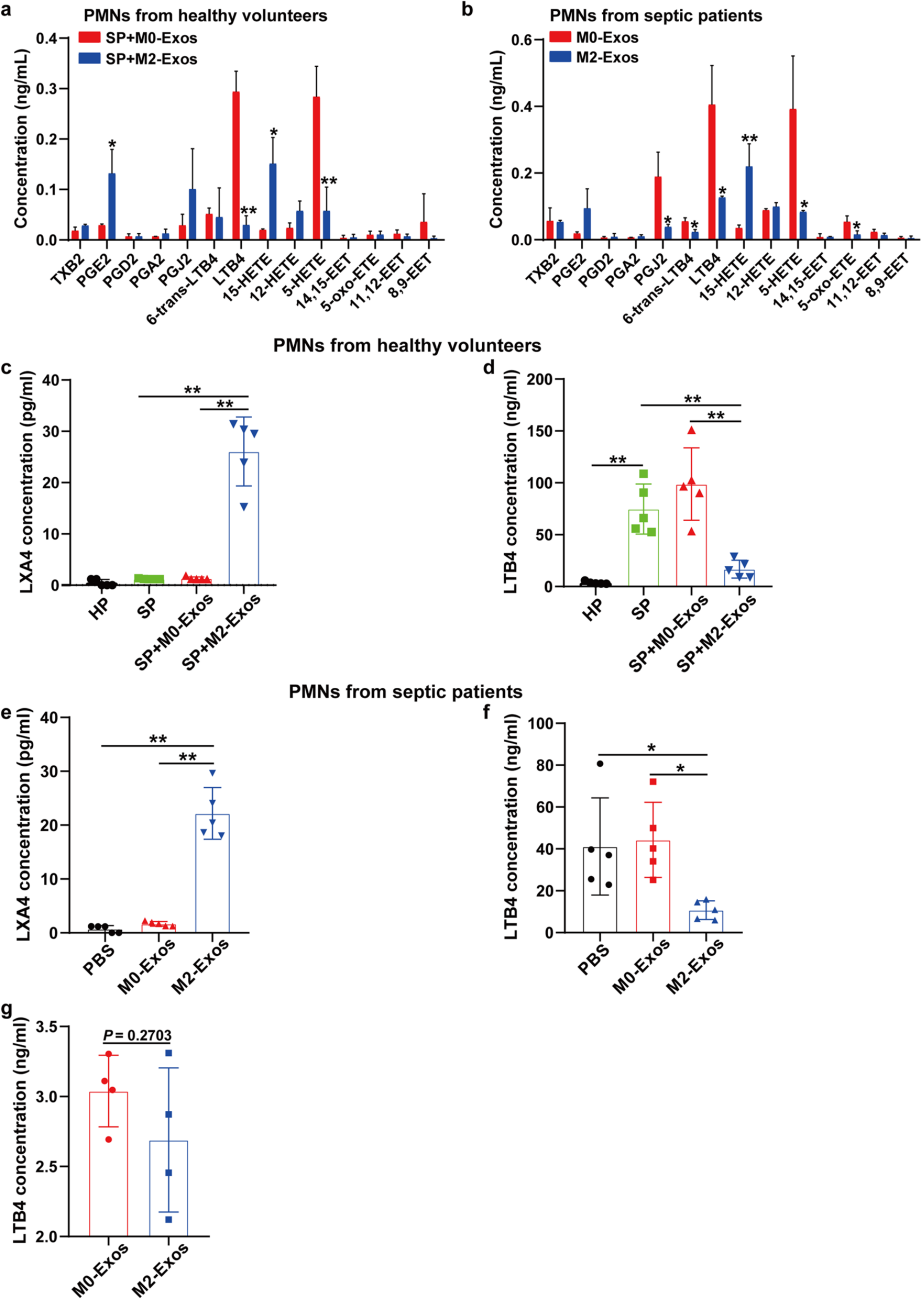
M2-Exos lead to lipid mediator class switching of PMNs during sepsis. **a**, **c** and **d** PMNs isolated from healthy volunteers were preactivated upon adding 20% septic plasma (SP) to the culture medium, and 20% plasma from healthy volunteers (HP) was used as a negative control. After 1 h, the culture medium was replaced with fresh medium, and PMNs were then cocultured with PBS/M0-Exos/M2-Exos (100 μg/mL) derived from PBMC-differentiated macrophages for 5 h. **b**, **e** and **f** PMNs from septic patients were directly cocultured with M0/M2-Exos (100 μg/mL) for 5 h after isolation. **a** and **b** Targeted metabolite analysis of eicosanoids in M0/M2-Exo-treated PMNs using liquid chromatography high-resolution mass spectrometry. LXA4 (**c** and **e**) and LTB4 (**d** and **f**) concentrations in the supernatant of cocultured PMNs were detected by ELISA kits. **g** LTB4 levels were compared in M0-Exos and M2-Exos from PBMC-differentiated macrophages by ELISA. One-way analysis of variance with Tukey’s multiple comparisons test (**c**–**f**) or Student’s t test (**a**, **b**, **g**) was used for the analysis. Graphs represent means ± standard deviations, n = 3–5; *P < 0.05, **P < 0.01 compared within two groups. TXB2, thromboxane B2; PGE2, prostaglandin E2; PGD2, prostaglandin D2; PGA2, prostaglandin A2; PGJ2, prostaglandin J2; 6-trans-LTB4,6-trans leukotriene B4; 15-HETE, 15-hydroxyeicosatetraenoic acid; 14,15-EET, 14,15-epoxy-5,8,11-eicosatrienoic acid; 5-oxo-ETE, 5-Oxo-6,8,11,14-eicosatetraenoic acid; 11,12-EET, 11,12-epoxy-5,8,14-eicosatrienoic acid; 8,9-EET, 8,9-epoxyeicosatrienoic acid

Next, we investigated how M2-Exos inhibited the migratory and NET formation ability of PMNs. For neutrophils, previous studies suggest that switching of eicosanoid biosynthesis from the predominantly proinflammatory lipid mediator LTB4 to the anti-inflammatory lipid LXA4 during acute exudate formation could “reprogram” exudate neutrophils to promote resolution—a process termed “lipid mediator class switching” [13]. However, whether this process also plays a role in inflammation resolution in sepsis remains unclear. Targeted metabolite analysis of arachidonic acid (AA)-derived eicosanoids in M0/M2-Exo-treated PMNs using liquid chromatography high resolution mass spectrometry revealed that compared to the M0-Exo group, the concentration of LTB4 was significantly lower (*P* < 0.05) and the concentration of 15-hydroxyeicosate-traenoic acid 15-HETE (LXA4 is produced by the metabolism of 15-HETE [23]) was significantly higher (*P* < 0.05) in M2-Exo-treated PMNs isolated from both healthy volunteers (Fig. 4a) and septic patients (Fig. 4b). Further ELISA in the present study also showed that septic plasma increased LTB4 production in the supernatant of PMNs isolated from healthy volunteers (Fig. 4d), while M2-Exos significantly increased the LXA4 concentration (Fig. 4c and e) and decreased the LTB4 concentration (Fig. 4d and f) in the supernatant of PMNs isolated from both healthy volunteers and septic patients. In addition, no significant difference in M0- or M2-Exo-carried LTB4 was found (Fig. 4g), nor was the LXA4 concentration in exosomes, which was below the detection limit of the ELISA kit (data not shown). All these results indicate that M2-Exos switched LTB4 to LXA4 production in PMNs during sepsis.

### M2-Exos inhibit PMN recruitment and NET formation through LXA4 upregulation

**Fig. 5:**
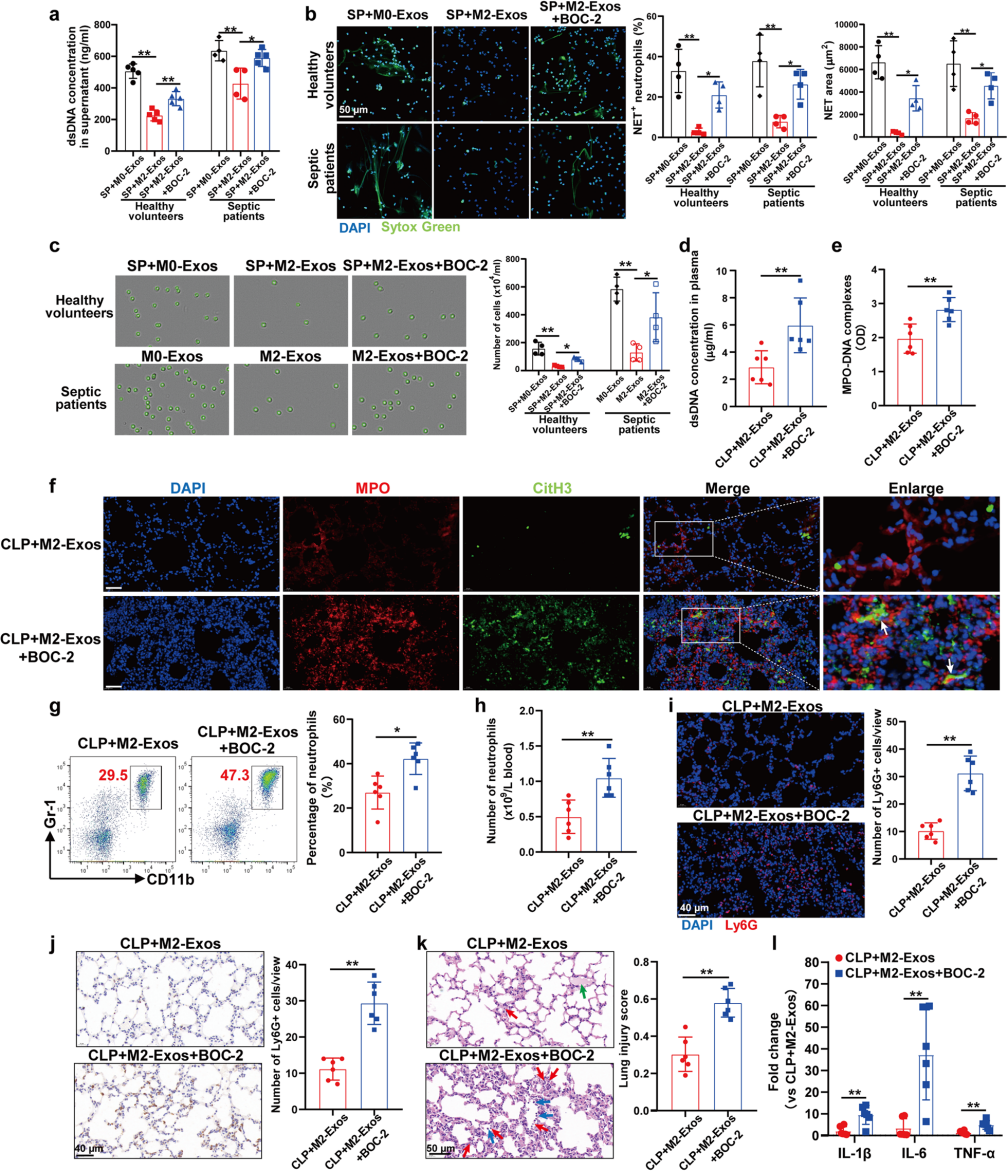
M2-Exos inhibit PMN recruitment and NET formation through LXA4 upregulation. **a** Ex vivo NET formation assay with PMNs isolated from healthy volunteers or septic patients activated by septic plasma (SP) and then cocultured with M0/M2-Exos (100 μg/mL) derived from PBMC-differentiated macrophages for 5 h with or without BOC-2 (10 µM). Quantification of dsDNA in the supernatant of cultured PMNs using PicoGreen fluorescent dye. **b** Typical images of NET formation using SYTOX Green (green). Scale bar, 50 μm. NET formation was quantified as the percentage of neutrophils forming NETs and the NET area per microscopic field. **c** Transwell analysis of PMN migration capacity isolated from healthy volunteers or septic patients. One-way analysis of variance with Tukey’s multiple comparisons test was used for the analysis. n = 4. **d**–**l** WT C57BL/6 mice were administered M2-Exos (300 μg/mouse) derived from mouse Raw264.7 macrophages via intraperitoneal injection 1 h after CLP. To block the LXA4 receptor, mice were treated with 50 µg/kg BOC-2 *i.p.* 30 min before CLP. **d** and **e** Quantification of dsDNA and circulating NET structures in the plasma of mice using PicoGreen fluorescent dye and MPO-DNA-ELISA, respectively. **f** Representative images showing the presence of NETs (MPO, red; citrullinated H3, green) in the lung tissues, as indicated by white arrows. Nuclei were counterstained with DAPI (blue). Scale bar, 40 μm. **g** Flow cytometry detection of the percentage of systemic circulating PMNs by staining with CD11b and Gr-1. **h** Absolute neutrophil number in peripheral blood. **i** and **j** Ly6G^+^ cells in the lung tissues were detected by immunofluorescence and immunohistochemistry. Scale bar, 40 μm. **k** Evaluation of lung histology by H&E staining (magnification × 400). Red arrows indicate neutrophils in the alveolar and interstitial space, blue arrows indicate alveolar macrophages, and green arrows indicate proteinaceous debris filling. Scale bar, 50 μm. Lung injury scores were assessed. **l** Detection of inflammatory cytokine mRNA (IL-1β, IL-6, TNF-α) expression in lung tissues by RT‒qPCR. Student’s t test was used for the analysis. Graphs represent means ± standard deviations, n = 6; *P < 0.05, **P < 0.01 compared within two groups

LXA4 is an important endogenous lipid that mediates the resolution of inflammation by functioning on the LXA4 receptor/formyl peptide receptor 2 (ALX/FPR2) [24]. To test whether upregulated LXA4 production mediated the inhibitory function of M2-Exos on PMNs, we chose BOC-2 (LXA4 receptor antagonist) to abrogate the effect of LXA4. First, we performed an in vitro coculture experiment and found that the migration and NET formation capacity of PMNs isolated from both healthy volunteers and septic patients inhibited by M2-Exos was reversed by BOC-2 treatment (Fig. 5a–c). To confirm the role of LXA4 in M2-Exo-induced inhibitory effects on PMN migration and NET formation, we also conducted experiments regarding the effects of BOC-2 and exogenous LXA4 on M0-Exo-treated PMNs. The results showed that BOC-2 did not influence the migration and NET formation capacity of M0-Exo-treated PMNs, while the addition of exogenous LXA4 to M0-Exo-treated PMNs inhibited NET formation and neutrophil migration capacity (Additional file 1: Fig. S6a-c).

To further investigate the LXA4-dependent action of M2-Exos in vivo, we coadministered M2-Exos and BOC-2 via intraperitoneal injection in the CLP model and found that BOC-2 increased the concentration of NET components in mouse plasma (Fig. 5d, e) and NET deposition in the lung (Fig. 5f) compared with the M2-Exo group. We also observed a significant increase in PMN recruitment to peripheral blood (Fig. 5g, h) and lung tissue (Fig. 5i, j) when septic mice were treated with M2-Exos + BOC-2 compared with that in the M2-Exo group. Consistent with the increased PMN recruitment and NET formation in the lung, the protective effects of M2-Exos against morphological changes and proinflammatory mediator production in the lung tissues were abolished by BOC-2 (Fig. 5k, l). In addition, coadministration of LXA4 and M0-Exos in the CLP model decreased NET formation and neutrophil migration to the circulation and lung tissues and alleviated lung injury compared to the M0-Exo only group, while BOC-2 treatment showed no difference from the M0-Exo group (Additional file 1: Fig. S6d-i). These data demonstrate that the M2-Exo-mediated reduction in CLP-induced PMN migration and NET formation was dependent on LXA4. However, it remains unclear how upregulated LXA4 alters PMN behaviour.

### LXA4 increased by M2-Exos downregulates CXCR2 and ROS expressions in PMNs

**Fig. 6:**
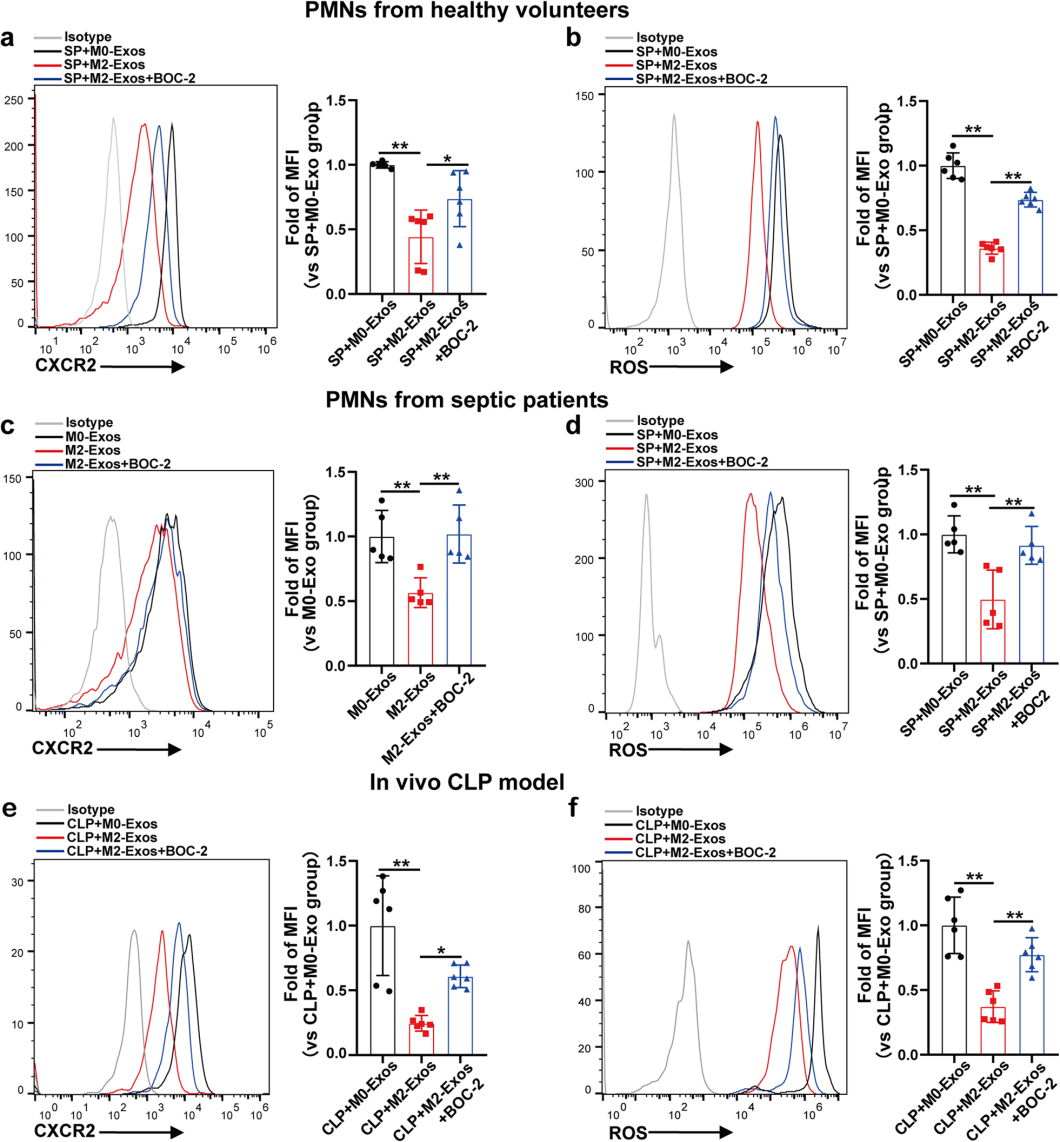
LXA4 increased by M2-Exos downregulates CXCR2 and ROS expressions in PMNs. PMNs isolated from healthy volunteers (**a** and **b**) or septic patients (**d**) were activated by septic plasma (SP) and then cocultured with M0/M2-Exos (100 μg/mL) derived from PBMC-differentiated macrophages for 5 h with or without BOC-2 (10 µM) incubation. **c** PMNs from septic patients were directly cocultured with M0/M2-Exos (100 μg/mL) for 5 h after isolation. CXCR2 (**a** and **c**) and ROS (**b** and **d**) expressions in cocultured PMNs were detected by flow cytometry. n = 4–5. **e** and **f** WT C57BL/6 mice were administered M0/M2-Exos (300 μg/mouse) derived from mouse Raw264.7 macrophages via intraperitoneal injection 1 h after CLP. To block the LXA4 receptor, mice were treated with 50 µg/kg BOC-2 *i.p.* 30 min before CLP. CXCR2 (**e**) and ROS (**f**) expressions in peripheral blood neutrophils were detected by flow cytometry. One-way analysis of variance with Tukey’s multiple comparisons test was used for the analysis. Graphs represent means ± standard deviations, n = 6; *P < 0.05, **P < 0.01 compared within two groups

Chemokine (C-X-C motif) receptor 2 (CXCR2) is a critical chemokine receptor responsible for neutrophil chemotaxis to infection sites [25]. In addition, CXCR2 signalling-induced and reactive oxygen species (ROS)-dependent NET formation has been demonstrated to be a therapeutic target in sepsis [22, 26]. Therefore, we postulated that LXA4 upregulation by M2-Exos could inhibit PMN activity by regulating CXCR2 and/or ROS expression. As expected, CXCR2 and ROS expressions in PMNs isolated from both healthy volunteers and septic patients were downregulated by M2-Exos compared with M0-Exos, which were reversed by BOC-2 (Fig. 6a–d). In addition, M2-Exo-induced CXCR2 and ROS downregulation in peripheral blood neutrophils was abrogated by BOC-2 in the CLP model (Fig. 6e, f). Exogenous addition of LXA4 decreased the expression of CXCR2 and ROS in PMNs both in vitro (Additional file 1: Fig. S7a-b) and in vivo (Additional file 1: Fig. S7c-d). All these data suggest that the effect of LXA4 upregulation by M2-Exos on PMNs might be mediated by downregulating CXCR2 and ROS expressions.

### M2-Exos promote LXA4 production in PMNs by increasing 15-LO expression

**Fig. 7:**
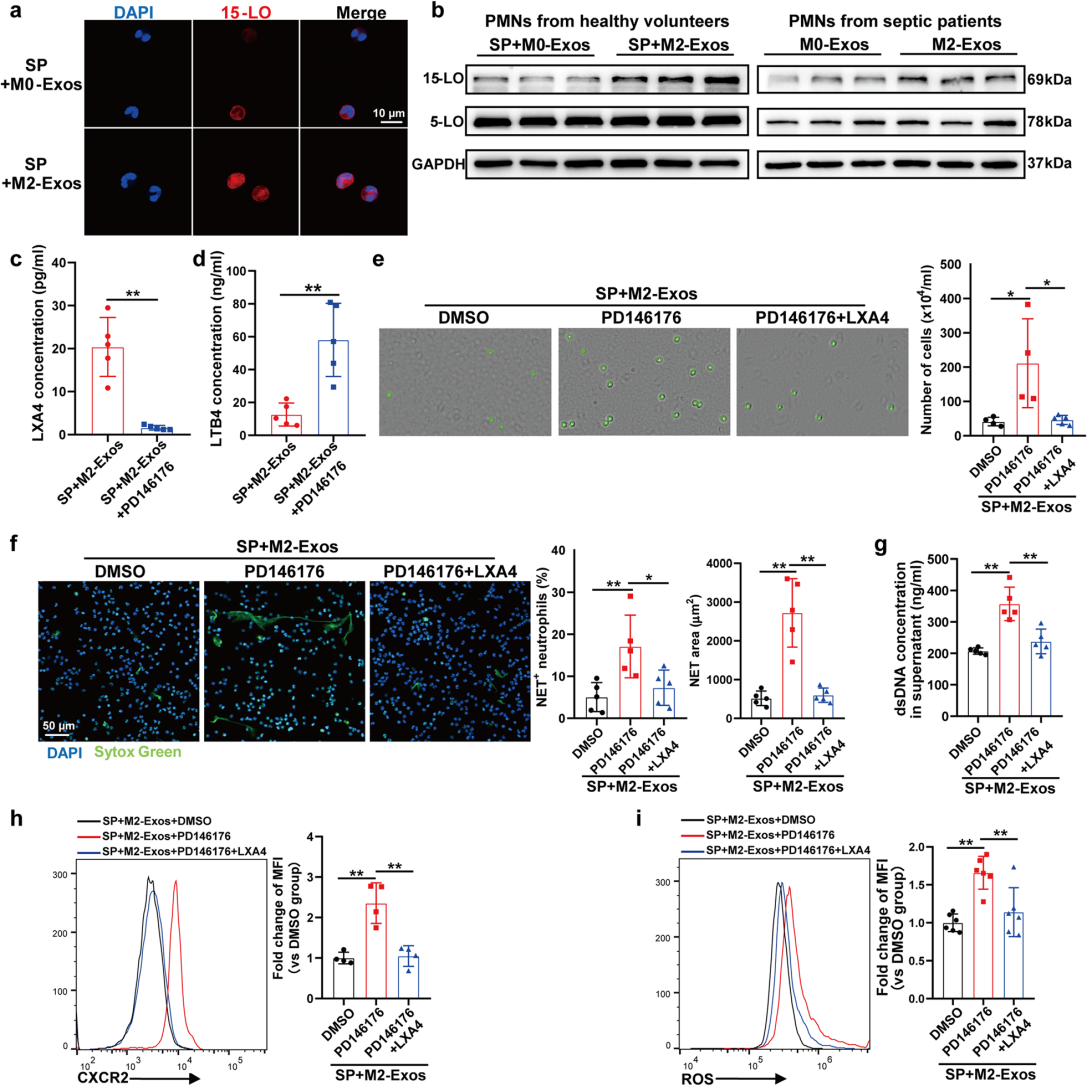
M2-Exos promote LXA4 production in PMNs by increasing 15-LO expression. **a**–**i** PMNs isolated from healthy volunteers were activated by septic plasma (SP) and then cocultured with M2-Exos (100 μg/mL) derived from PBMC-differentiated macrophages for 5 h with or without PD146176 (1 µM) and LXA4 (100 nM), as indicated in the figures. DMSO was used as a negative control. **a** Representative images of 15-LO in PMNs detected by immunofluorescence. (**b** left panel) 15-LO and 5-LO expressions in PMNs were detected by Western blot. (**b** right panel) PMNs isolated from septic patients were cocultured with M0/M2-Exos (100 μg/mL) derived from PBMC-differentiated macrophages for 5 h. 15-LO and 5-LO expressions in PMNs were detected by Western blot. **c** and **d** LXA4 and LTB4 concentrations in the supernatant of PMNs were detected by ELISA. **e** Transwell analysis of neutrophil chemotaxis towards IL-8. **f** Typical images of NET formation using SYTOX Green (green). Scale bar, 50 μm. NET formation was quantified as the percentage of neutrophils forming NETs and the NET area per microscopic field. **g** Quantification of dsDNA in the supernatant of cultured PMNs using PicoGreen fluorescent dye. Flow cytometry detection of CXCR2 (**h**) and ROS (**i**) expressions in cocultured PMNs. Student’s t test (**c** and **d**) or one-way analysis of variance with Tukey’s multiple comparisons test (**e**–**i**) was used for the analysis. Graphs represent means ± standard deviations, n = 4–6; *P < 0.05, **P < 0.01 compared within two groups

We next investigated how M2-Exos increased LXA4 production in PMNs. Previous studies have shown that arachidonate 15-lipoxygenase (15-LO) activity, in combination with arachidonate 5-lipoxygenase (5-LO), can produce lipoxins, and 5-LO activity alone forms leukotrienes [27]. As shown in Fig. 4a and b, M2-Exos increased the 15-LO product, 15-HETE concentration in PMNs, indicating that higher 15-LO activity was induced following M2-Exo treatment. Additionally, we found that M2-Exos increased 15-LO expression in PMNs isolated from both healthy volunteers and septic patients, while the expression of 5-LO remained unchanged (Fig. 7a, b). Treatment with PD146176, a specific 15-LO inhibitor, significantly decreased the LXA4 concentration and increased the LTB4 concentration in the supernatant of M2-Exo-treated PMNs (Fig. 7c, d). PD146176 also increased the migration and NET formation capacity of M2-Exo-treated PMNs, and the addition of exogenous LXA4 following 15-LO inhibition abrogated the effect of PD146176 (Fig. 7e–g). Flow cytometry showed that CXCR2 and ROS expressions in M2-Exo-treated PMNs were increased after PD146176 treatment, which were reversed by LXA4 addition (Fig. 7h, i). All these results indicate that M2-Exos promoted LXA4 production by increasing 15-LO expression in PMNs.

### Exosomal PGE2 from M2 macrophages is necessary for 15-LO upregulation and PMN inhibition

**Fig. 8:**
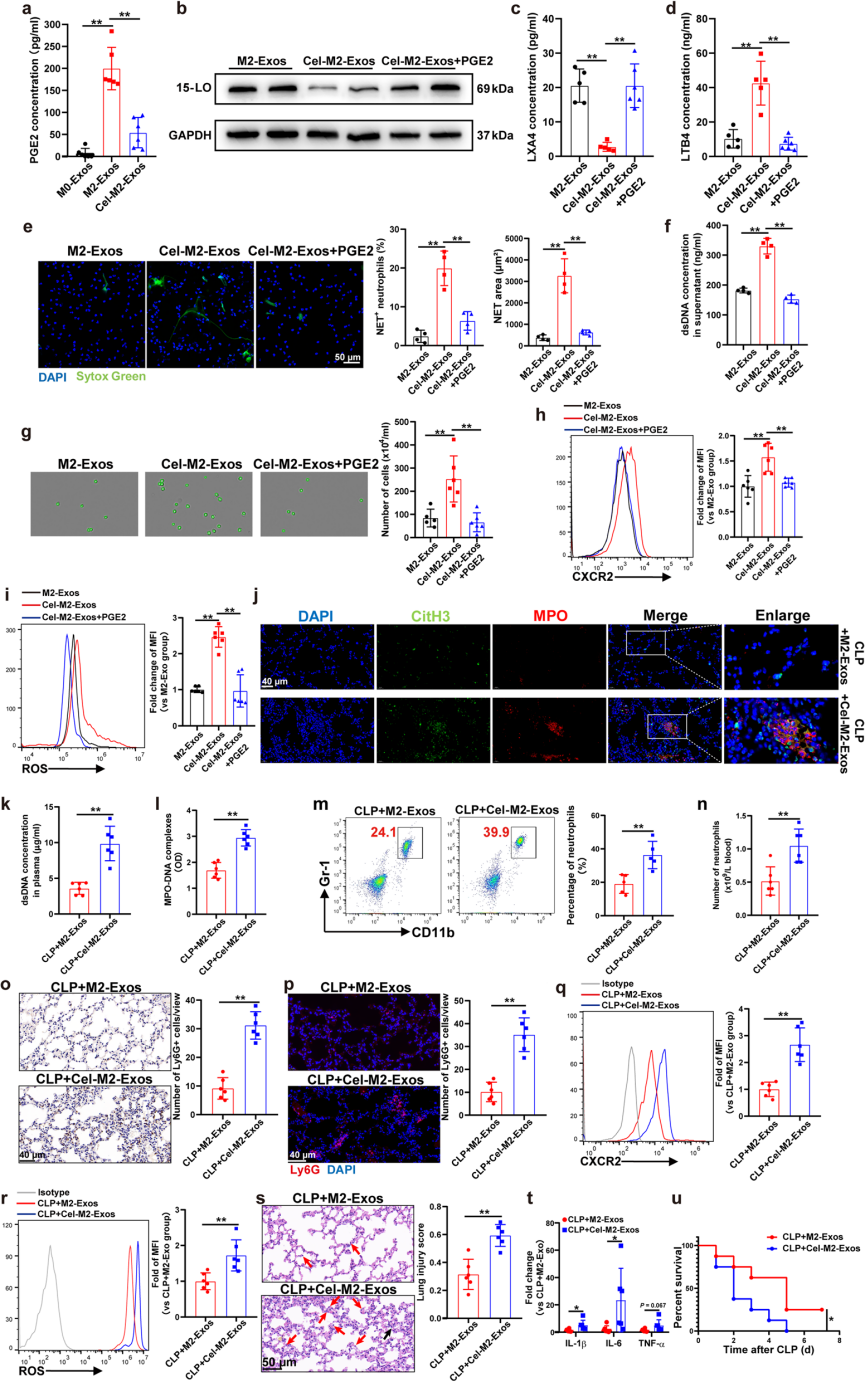
Exosomal PGE2 from M2 macrophages is necessary for 15-LO upregulation and PMN inhibition. **a** PGE2 levels in M0-Exos/M2-Exos/exosomes from celecoxib (20 µM)-treated PBMC-differentiated M2 macrophages (Cel-M2-Exos) were examined by ELISA. PMNs isolated from healthy volunteers were activated by septic plasma (SP) and then cocultured with M2-Exos/Cel-M2-Exos/Cel-M2-Exos + PGE2 (100 nM) for 5 h. **b** Immunoblot analysis of 15-LO in PMNs. **c** and **d** LXA4 and LTB4 concentrations in the supernatant of PMNs were detected by ELISA. **e** Typical images of NET formation using SYTOX Green (green). Scale bar, 50 μm. NET formation was quantified as the percentage of neutrophils forming NETs and the NET area per microscopic field. **f** Quantification of dsDNA in the supernatant of cultured PMNs using PicoGreen fluorescent dye. **g** Transwell analysis of neutrophil chemotaxis towards IL-8. Flow cytometry detection of CXCR2 (**h**) and ROS (**i**) expressions in cocultured PMNs. One-way analysis of variance with Tukey’s multiple comparisons test was used for the analysis. n = 4–6. **j**–**u** WT C57BL/6 mice were administered M2-Exos/Cel-M2-Exos (300 μg/mouse) derived from mouse Raw264.7 macrophages via intraperitoneal injection 1 h after CLP. **j** Representative images showing the presence of NETs (MPO, red; citrullinated H3, green) in the lung tissues, as indicated by white arrows. Nuclei were counterstained with DAPI (blue). Scale bar, 40 μm. **k** and **l** Quantification of dsDNA and circulating NET structures in the plasma of mice using PicoGreen fluorescent dye and MPO-DNA-ELISA, respectively. **m** Flow cytometry detection of the percentage of systemic circulating neutrophils by staining with CD11b and Gr-1. **n** Absolute neutrophil number in peripheral blood. **o** and **p** Ly6G^+^ cells in the lung tissues were detected by immunohistochemistry and immunofluorescence. Scale bar, 40 μm. CXCR2 (**q**) and ROS (**r**) expressions in peripheral blood neutrophils were detected by flow cytometry. **s** Evaluation of lung histology by H&E staining (magnification × 400). Red arrows indicate neutrophils in the alveolar and interstitial space, and black arrows indicate thickening of the alveolar walls. Scale bar, 50 μm. Lung injury scores were assessed. **t** Detection of inflammatory cytokine mRNA (IL-1β, IL-6, TNF-α) expression in lung tissues by RT‒qPCR. Student’s t test was used for the analysis. Graphs represent means ± standard deviations, n = 6. **u** Survival rate of CLP mice treated with M2-Exos or Cel-M2-Exos (n = 8); the log-rank test was used for the analysis. *P < 0.05, **P < 0.01 compared within two groups

A recent study reported that PGE2 could promote resolution of inflammation by switching lipid mediator biosynthesis and guiding neutrophil phenotype alteration [28]. As indicated by our targeted metabolite analysis results, the PGE2 concentration tended to be higher in M2-Exo-treated PMNs than in the M0-Exo-treated PMNs (Fig. 4a, b). We therefore sought to determine whether PGE2 could be transferred through M2-Exos and whether PGE2 in M2-Exos mediated its inhibitory effect on PMNs. First, we showed that the PGE2 level in M2-Exos was significantly higher than that in M0-Exos (Fig. 8a). Septic plasma (SP) treatment slightly increased PGE2 level in the supernatant of cultured PMNs compared to the plasma from healthy control-treated PMN group, and M2-Exo treatment significantly increased PGE2 level compared to that in the M0-Exo group (Additional file 1: Fig. S8c). To delete PGE2 in M2-Exos, we blocked PGE2 production by celecoxib, a selective cyclooxygenase-2 (COX-2) inhibitor. The PGE2 level in exosomes from celecoxib-treated M2 macrophages (Cel-M2-Exos) was significantly lower than that in M2-Exos (Fig. 8a). As expected, 15-LO expression in Cel-M2-Exo-treated PMNs was lower than that in M2-Exo-treated PMNs, and the addition of exogenous PGE2 to Cel-M2-Exos increased 15-LO expression in PMNs (Fig. 8b), indicating that 15-LO upregulation in PMNs following M2-Exo treatment was dependent on PGE2. Additionally, Cel-M2-Exo-treated PMNs showed impaired lipid mediator class switching, while the addition of PGE2 mimicked the effects of M2-Exos on LXA4 and LTB4 alteration (Fig. 8c, d).

Additionally, the in vitro coculture experiment showed that NET formation (Fig. 8e, f), neutrophil migration capacity (Fig. 8g), CXCR2 (Fig. 8h) and ROS expressions (Fig. 8i) of PMNs were all increased in the Cel-M2-Exo group compared with the M2-Exo group, while supplementation with PGE2 in the culture medium reversed the effects of Cel-M2-Exos on PMNs.

To confirm the results obtained from the in vitro experiment, we administered M2-Exos or Cel-M2-Exos *i.p.* one hour following CLP, we found an increasement in NET formation (Fig. 8j–l), trafficking of neutrophils from bone marrow to the circulation (Fig. 8m, n) and lung tissues (Fig. 8o, p), and increased expression of CXCR2 (Fig. 8q) and ROS (Fig. 8r) in peripheral blood neutrophils following Cel-M2-Exo administration compared with M2-Exo administration. In addition, the lung injury score (Fig. 8s), proinflammatory mediator production in the lung tissue (Fig. 8t) and mortality rate (Fig. 8u) in the Cel-M2-Exo group were all significantly higher than those in the M2-Exo group.

To further clarify the protective role of PGE2 in M2-Exos, we conducted experiments regarding the direct effects of PGE2 on SP/SP + M0-Exo-treated PMNs. First, the viability of PMNs was examined, and the results showed that PGE2 (100 nM) treatment did not influence the apoptotic rate of SP/SP + M0-Exo-treated PMNs (Additional file 1: Fig. S9a). PGE2 increased 15-LO expression in both SP and SP + M0-Exo-treated PMNs (Additional file 1: Fig. S9b). Additionally, PGE2 showed the ability to switch LTB4 to LXA4 production (Additional file 1: Fig. S9c-d). NET formation (Additional file 1: Fig. S9e-f), neutrophil migration capacity (Additional file 1: Fig. S9g), CXCR2 (Additional file 1: Fig. S9h) and ROS expressions (Additional file 1: Fig. S9i) in PMNs were all decreased following PGE2 treatment. The direct effects of PGE2 on sepsis-related ALI in CLP mice were also determined, and the results showed a reduction in NET formation (Additional file 1: Fig. S10a-c), trafficking of neutrophils from bone marrow to the circulation (Additional file 1: Fig. S10d) and lung tissues (Additional file 1: Fig. S10e), and decreased expression of CXCR2 and ROS (Additional file 1: Fig. S10f-g) in peripheral blood neutrophils. In addition, the lung injury score and proinflammatory mediator production in the lung tissues in the M0-Exos + PGE2 group were all significantly lower than those in the M0-Exo alone group (Additional file 1: Fig. S10h-i).

Collectively, these data suggest that exosomal PGE2 derived from M2 macrophages increased 15-LO expression in PMNs to upregulate LXA4 production and then modulated neutrophil migratory ability and NET formation capacity.

### Exosomal PGE2 from M2 macrophages functions on the EP4 receptor of PMNs

PGE2 has been proven to signal in vivo through the EP4 receptor of PMNs and then increase 15-LO expression [14]. To further test whether exosomal PGE2 from M2 macrophages modulated the neutrophil phenotype by functioning on the EP4 receptor, we used a specific antagonist (E7046) to block the binding of PGE2 to its receptor EP4. As shown in Additional file 1: Fig. S11a, 15-LO expression was significantly lower in the M2-Exos + E7046 group than in the M2-Exo group. The M2-Exo-induced alteration of LXA4 and LTB4 was blocked following E7046 treatment (Additional file 1: Fig. S11b-c). Treatment with E7046 also increased NET formation (Additional file 1: Fig. S11d-e), neutrophil migration capacity (Additional file 1: Fig. S11f), CXCR2 (Additional file 1: Fig. S11g) and ROS expressions (Additional file 1: Fig. S11h) in PMNs, while LXA4 addition abrogated the effects of E7046 on PMNs.

In addition, in vivo experiments using the CLP model demonstrated that E7046 increased NET deposition in the lung (Additional file 1: Fig. S12a) and the concentration of NET components in mouse plasma (Additional file 1: Fig. S12b-c) compared with the M2-Exo only group. We also observed a significant increase in PMN recruitment to peripheral blood (Additional file 1: Fig. S12d) and lung tissues (Additional file 1: Fig. S12e) and increased expression of CXCR2 and ROS (Additional file 1: Fig. S12f-g) in peripheral blood neutrophils when CLP mice were treated with M2-Exos + E7046 compared with the M2-Exo only group. The protective effects of M2-Exos against morphological changes and proinflammatory mediator production in the lung tissues of CLP mice were also abolished by E7046 (Additional file 1: Fig. S12h-i).

## Discussion

**Fig. 9:**
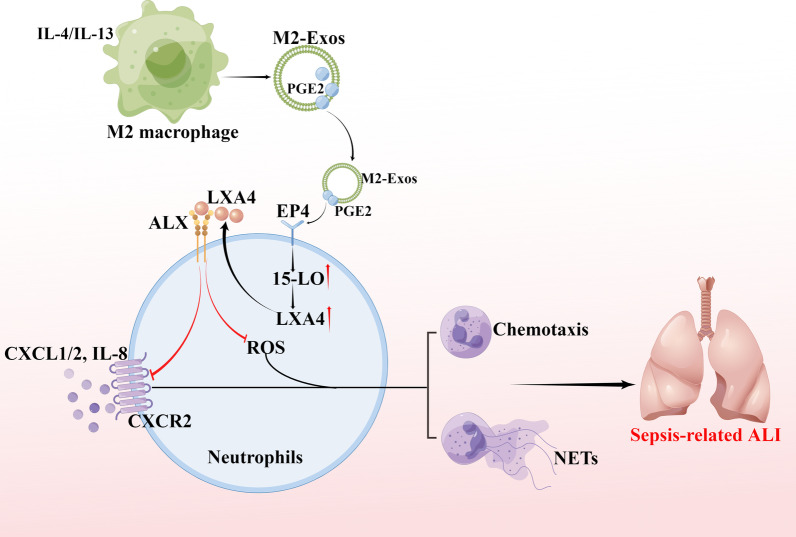
Working model. Our data demonstrated that exosomal PGE2 from M2 macrophages functions on the EP4 receptor to increase 15-LO expression in PMNs, upregulate LXA4 production to downregulate PMN CXCR2 and ROS expressions, inhibit the capacity of PMN migration and NET formation, alleviate lung injury, and reduce mortality in sepsis. The graph was drawn on the Figdraw online website (Export ID: RYUYR79822)

In this study, we investigated the effect of exosomes secreted by M2 macrophages on PMNs in sepsis-induced ALI by using a CLP murine model and in vitro coculture experiments. We demonstrated that M2-Exos could alleviate lung injury and reduce the mortality rate during sepsis. We further suggested that the underlying mechanism was that exosomal PGE2 from M2 macrophages upregulated LXA4 production in PMNs by increasing 15-LO expression to modulate the capacity of PMN migration and NET formation, possibly by downregulating PMN CXCR2 and ROS expressions (Fig. 9).

In the early stage of sepsis, PMNs are thought to be the primary innate immune cells that cause damage to host tissues [29]. An imbalance in the number, recruitment or functionality of PMNs results in an excessive inflammatory response and life-threatening critical syndromes following the onset of sepsis, making PMNs attractive therapeutic targets [3]. Accumulating evidence documents the phenotypic and functional versatility of PMNs, and their diverse roles in innate and adaptive immune responses provide important cues for the development of PMN-targeting therapies [30]. However, currently, an ideal therapeutic strategy that could prevent or reverse PMN-mediated tissue injury without impairing their ability to control microbial invasion is still lacking.

Macrophages are also well known to be key mediators in determining the outcome of the inflammatory response, and the interaction between PMNs and macrophages has gained increasing interest in the sepsis research field. Our previous studies demonstrated that alveolar macrophage-derived exosomes induced PMNs to undergo necroptosis [31], and PMN-derived exosomes promoted macrophage M1 polarization and pyroptosis during sepsis [7]. Other studies have also shown that macrophage-derived exosomes recruit and activate PMNs in sepsis [32], indicating that macrophage-derived exosomes could serve as a therapeutic target for PMN-mediated tissue injury during sepsis.

M2-Exos have been studied in different disease settings, such as spinal cord damage [33], myocardial ischaemia‒reperfusion [10], atherosclerosis [34], and inflammatory pain [35], all showing promising therapeutic effects. However, it is unknown whether M2-Exos could induce anti-inflammatory activities in sepsis-associated tissue injury. Our study first showed that M2-Exos could clearly alleviate sepsis-induced lung injury and improve survival without significantly affecting bacterial load in the lung, suggesting that M2-Exos would be an appropriate treatment for sepsis. Further in vitro coculture experiments were conducted using plasma from septic patients to activate PMNs according to previous studies [22, 36, 37]. Many mediators enriched in septic plasma have been demonstrated to activate PMNs and induce NET formation, including IL-8 [22], TNF-α [38], IL-1β [39], platelet-derived exosomes [18] and the bacterial components LPS and PGN [40, 41]. However, these mediators, especially lipid mediators, in septic plasma may influence the results of our current in vitro experiments. Therefore, in our experimental design, after a 1-h pretreatment with septic plasma, the culture medium was replaced with fresh medium, and PMNs were then cocultured with PBS/M0-Exos/M2-Exos (100 μg/mL) derived from PBMC-differentiated macrophages for an additional 5 h. We found an immunosuppressive effect of M2-Exos on PMNs from both healthy volunteers and septic patients ex vivo, also highlighting that M2-Exos would be a promising therapeutic candidate for PMN-mediated tissue injury during sepsis and help us better understand the endogenous mechanisms for the resolution of inflammation.

A growing body of evidence indicates that resolution of inflammation is an active process involving the production of lipid-derived specialized proresolving mediators (SPMs), which include lipoxins, resolvins, protectins, and maresins [42]. SPMs actively stimulate cardinal signs of resolution, namely, cessation of leukocytic infiltration, counterregulation of proinflammatory mediators, and uptake of apoptotic PMNs and cellular debris [30]. The biosynthesis of these resolution-phase mediators in sensu stricto is initiated during lipid mediator class switching, in which the classic initiators of acute inflammation, prostaglandins and leukotrienes, switch to produce SPMs [43]. Previous studies have proven that lipid mediator class switching plays a critical role in determining the neutrophil phenotype, altering neutrophil migratory behaviour [13, 14]. Our data also showed that M2-Exos promoted lipid mediator class switching from LTB4 to LXA4 in PMNs. LTB4 is a product of the 5-LO pathway and is known to strongly initiate and amplify PMN chemotaxis as well as the release of granule products and superoxide anions [44]. While LTB4-stimulated PMN functional responses are opposed by LXA4, a distinct class of 15-LO-derived eicosanoids, signals through ALX/FPR2 to limit neutrophil trafficking and lifespan and to promote efferocytosis, emerged as master regulator of neutrophil responses and fates. Therefore, the use of LXA4 to treat sepsis has gained great attention, and consistent with our results, mounting evidence has shown that LXA4 limits leukocyte recruitment and relieves sepsis-related ALI [45, 46].

Our study showed that M2-Exos did not influence the bacterial load in lung tissues, even with the downregulation of PMN migration and NET formation. A previous study [47] demonstrated that LXA4 treatment decreased PMN migration to the peritoneum but augmented blood and peritoneal PMN phagocytic ability in a CLP-induced model of sepsis. Further ex vivo experiments also proved that LXA4 (1 nM) increased phagocytosis in blood PMNs without affecting apoptosis. This phenotype may partly explain our result, suggesting that M2-Exo-induced LXA4 upregulation could reduce PMN migration and increase their bacterial clearance function.

PMN recruitment from bone marrow to infectious tissues is critical for early innate responses, which are mainly triggered by interactions between chemokines and chemokine receptors [48]. The chemokine receptor CXCR2 is largely responsible for driving PMN migration during bacterial infection and inflammation [49]. In addition, a recent study [22] showed that inhibition of CXCR1/2 by reparixin reduced NET formation, multiorgan injury, and mortality in septic mice without impairing bacterial clearance, which highlights that CXCR1/2 signalling-induced NET formation is a therapeutic target in sepsis. In this study, following treatment with M2-Exos, we observed reduced CXCR2 expression in PMNs. Blocking LXA4 function reversed the expression of CXCR2, suggesting that M2-Exos upregulated LXA4 to reduce CXCR2 expression in PMNs. In accordance with our results, LXA4 was shown to inhibit chemokine signalling in PMNs in vitro, and chemokine receptor desensitization is a strong candidate for explaining how LXA4 alters the PMN migration pattern, leading to inflammation resolution [50].

Neutrophil ROS production plays a critical role in antibacterial host defence, although an uncontrolled continuous oxidative burst response can be detrimental [51]. In this study, we also demonstrated that treating PMNs from both healthy volunteers and septic patients with M2-Exos reduced the excessive ROS production elicited by septic plasma. These findings are consistent with a previous study [52], which demonstrated that LXA4 treatment reduced *Porphyromonas gingivalis*-induced leukocyte ROS production in human whole blood. As elevated ROS production may promote leukocyte infiltration and NET formation, M2-Exo-mediated attenuation of PMN function may be partly due to reduced ROS production.

Previously, PGE2 was shown to be a well-known proinflammatory lipid mediator produced by prostaglandin E synthase in leukocytes. Currently, accumulating evidence indicates that PGE2 has both anti- and proinflammatory effects dependent on the timing of its production and concentration [53]. A previous study suggested that PMN exposure to PGE2 induced the phenotype switch from LTB4 production to lipoxin production, which marks the resolution phase [14]. Our data also showed that the level of PGE2 in M2-Exos was significantly higher than that in M0-Exos, which begs the question of whether exosomal PGE2 from M2 macrophages could alter the lipid mediator class in PMNs. COX-2 is an inducible enzyme that can convert arachidonic acid (AA) into PGE, and the level of PGE2 is typically used as an indicator of COX-2 activity [54]. Therefore, we used the COX-2 inhibitor celecoxib to delete PGE2 expression in M2-Exos according to a previously published paper [55]. The results showed that M2 macrophages secreted PGE2 through exosomes to switch the lipid mediator class in PMNs. It has been shown that exosomes expressing PGE2 can interact with PGE2 receptors (EP2, EP4) on dendritic cells, leading to CD73 production by the latter [56], which is consistent with our result that inhibition of the EP4 receptor significantly abrogated the effect of M2-Exos on PMNs.

## Conclusion

This study reveals a hitherto unknown role of exosomal PGE2 derived from M2 macrophages in increasing 15-LO expression in PMNs, upregulating LXA4 production to downregulate PMN CXCR2 and ROS expressions, inhibiting PMN migration and NET formation, alleviating lung injury, and reducing mortality in sepsis. These findings may add a new element to macrophage exosome-PMN crosstalk, and M2-Exos may therefore be a better treatment to target PMN-mediated tissue injury in patients with sepsis.

## Supplementary Information

**Additional file 1. **Supplementary table and figures.

**Additional file 2. **Supplementary methods.

## Data Availability

The datasets generated and analyzed during the current study are available from the corresponding author on reasonable request.

## Abbreviations

PMNs: Polymorphonuclear neutrophils
NET: Neutrophil extracellular trap
M2-Exos: M2 macrophage-derived exosomes
ALI: Acute lung injury
CLP: Cecal ligation and puncture
H&E: Hematoxylin and eosin
LTB4: Leukotriene B4
LXA4: Lipoxin A4
15-LO: 15-Lipoxygenase
PGE2: Prostaglandin E2
CXCR2: Chemokine (C-X-C motif) receptor 2
ROS: Reactive oxygen species
MODS: Multiple organ dysfunction syndrome
ARDS: Acute respiratory distress syndrome
5-LO: 5-Lipoxygenase
PBMCs: Peripheral blood mononuclear cells
RPMI: Roswell park memorial institute
FBS: Fetal bovine serum
rhM-CSF: Recombinant human macrophage-colony stimulating factor
IL-4: Interleukin 4
SP: Septic plasma
HP: Plasma from healthy volunteers
MPO: Myeloperoxidase
WT: Wild type
*i.p.*: Intraperitoneally
CFU: Colony forming units
IHC: Immunohistochemistry
IF: Immunofluorescence
PBS: Phosphate buffer saline
CitH3: Citrullinated histone H3
ELISA: Enzyme-linked immunosorbent assay
TNF-α: Tumor necrosis factor-α
AA: Arachidonic acid
15-HETE: 15-Hydroxyeicosate-traenoic acid
LPS: Lipopolysaccharides
PGN: Peptidoglycan
COX-2: Cyclooxygenase-2
SPMs: Specialized pro-resolving mediators
