# Supplementary material for: Exosomal PGE2 from M2 macrophages inhibits neutrophil recruitment and NET formation through lipid mediator class switching in sepsis
Source: J Biomed Sci. 2023 Aug 2;30:62. doi: 10.1186/s12929-023-00957-9 (PMC10394797; doi:10.1186/s12929-023-00957-9)
Supplement: Supplementary file 1 — Additional file 1. Supplementary table and figures. [file 12929_2023_957_MOESM1_ESM.pdf]

Supplementary Table

Table 1 Demographic data of the included septic patients

**Characteristics**	**Septic patients (N = 25)**
Male sex, n (%)	21 (84)
Age, years	64 (46.5-77)
Mortality at 28 days, n (%)	6 (24)
Comorbidities, n (%)	
Arterial hypertension	5 (20)
Diabetes mellitus	3 (12)
Source of sepsis, n (%)	
Urinary	4 (16)
Abdominal	20 (80)
Lung	1 (4)
Hemodynamic data	
Heart rate/min	93 (76-102.5)
Mean arterial pressure, mmHg	81.0 ± 15.4
Norepinephrine dosage, μg/kg/min	0.04 (0-0.13)
Ventilatory data	
Respirate rate/min	12 (12-12.5)
PaCO2 (mmHg)	43.8 ± 9.4
PaO2/FIO2 (Kpa)	307.3 ± 110.8
Use of mechanical ventilation, n (%)	21 (84)
Hematologic and inflammatory data	
Neutrophils, 10^9/L	14.8 ± 6.1
Hemoglobin, g/dL	102.7 ± 21.0
Platelets, 10^9/L	137 (110.5-220)
Lactate, mmol/L	1.7 (1-2.3)
CRP, mg/dL	140.9 (58.4-249.7)
Procalcitonin, ng/mL	4.1 (0.5-24.9)
SOFA score	4 (3-5)

CRP: C-reactive protein, SOFA: Sequential Organ Failure Assessment.

Data are expressed as number (%), mean ± SD, or median (25th-75th percentile).

Supplementary Figures

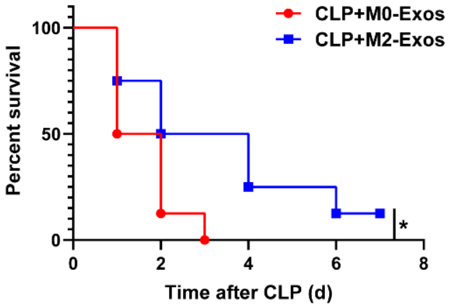

Figure S1 M2-Exos improve survival of septic mice. WT C57BL/6 mice were administered with M0-Exos or M2-Exos (150 μg/mouse) derived from mouse Raw264.7 macrophages via intraperitoneal injection 1 hour after CLP. Survival rate of CLP mice with M0-Exo or M2-Exo treatment (n = 8) and log-rank test was used for the analysis. *$P < 0.05$ compared within two groups.

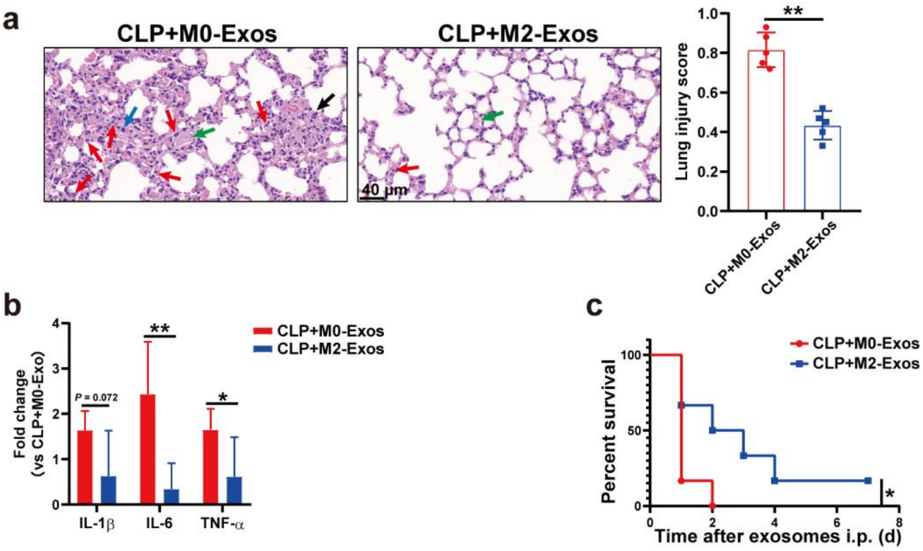

Figure S2 M2-Exos given 1 day after CLP reduce lung injury and improve survival of septic mice. WT C57BL/6 mice were administered with M0-Exos or M2-Exos (300 μg/mouse) derived from mouse Raw264.7 macrophages intraperitoneally 1 day after CLP. Lung tissues were collected 24 h after exosome administration. (**a**) Evaluation of lung histology by H&E staining (magnifcation×400). Red arrows indicate neutrophils in the alveolar and interstitial space, blue arrows indicate alveolar macrophages, green arrows indicate proteinaceous debris filling, and black arrows indicate thickening of the alveolar walls. Scale bar, 40 μm. Lung injury scores were assessed. (**b**) Detection of inflammatory cytokine mRNA (IL-1β, IL-6, TNF-α) expression in the lung tissues by RT-qPCR. Student's t test was used for the analysis. (**c**) Survival rate of CLP mice with M0-Exo or M2-Exo treatment (n = 6) and log-rank test was used for the analysis. Graphs represent means ± standard deviations, n ≥ 3; *$P < 0.05$, **$P < 0.01$ compared within two groups.

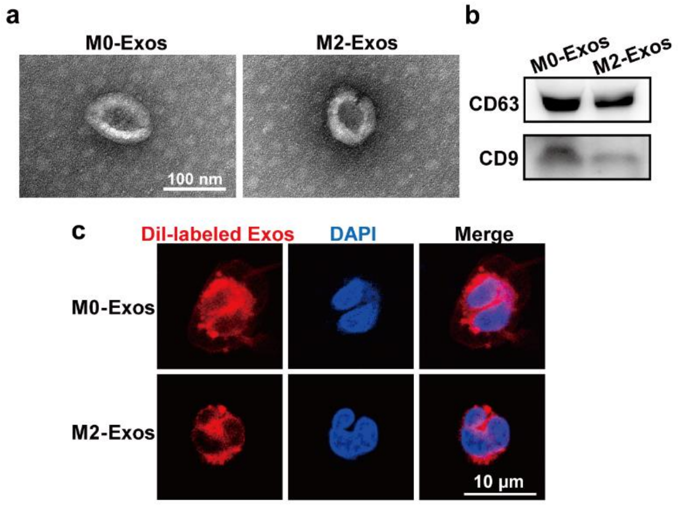

Figure S3 The characterization of M0/M2-Exos derived from PBMC-differentiated macrophages. (**a**) Electron micrograph of exosomes showed a round, cup-shaped morphology, with a diameter about 100 nm. Scale bar, 100 nm. (**b**) CD63 and CD9 protein expressions in exosomes were quantified by Western blot loaded with equal amounts of exosome protein (40 μg). (**c**) Exosomes were stained with Dil cell-labeling solutions. Immunofluorescence images show PMNs incubated with Dil-labeled exosomes (red) for 5 h. Nuclei were counterstained with DAPI (blue).

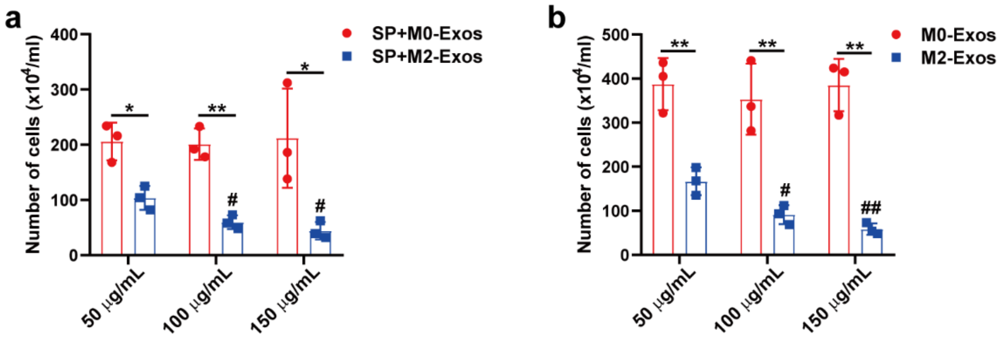

Figure S4 M2-Exos inhibit PMN recruitment toward IL-8. (**a**) PMNs from healthy volunteers were activated by septic plasma, and then cocultured with M0/M2-Exos (50-150 μg/mL) derived from PBMC-differentiated macrophages. After 5 h, PMNs were collected for migration capacity analysis with IL-8 as a chemokine. After 2-h incubation, cells in the lower chamber were collected and counted under a microscope. (**b**) PMNs isolated from septic patients were directly co-cultured with M0/M2-Exos (50-150 μg/mL) derived from PBMC-differentiated macrophages for 5 h after isolation. Then, PMNs were transferred for transwell assay. Graphs represent means ± standard deviations, n = 3; *$P < 0.05$, **$P < 0.01$ compared between the indicated two groups; $^{\#}P < 0.05$, $^{\#\#}P < 0.01$ compared between the indicated group and 50 μg/mL group.

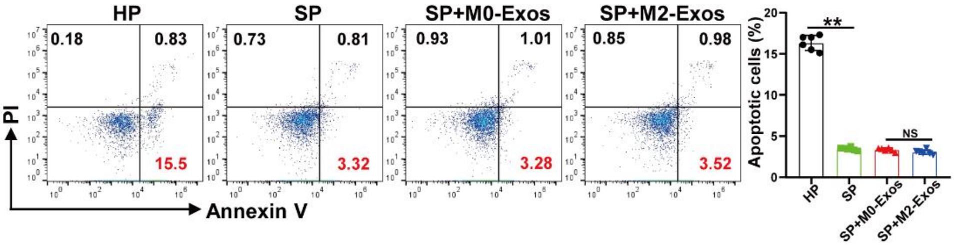

Figure S5 PMNs from healthy volunteers were activated by septic plasma (SP), and then cocultured with M0/M2-Exos (100 μg/mL) derived from PBMC-differentiated macrophages for 5 h. Plasma from healthy volunteers (HP) was used as negative control. Representative flow cytometry plots of Annexin V/PI staining of PMNs, and analysis of Annexin V positive/PI negative-stained cells by apoptotic cells. One-way analysis of variance with Tukey’s multiple comparisons test was used for the analysis. Graphs represent means ± standard deviations, n = 6; *P < 0.05, **P < 0.01 compared within two groups. NS, not significant.

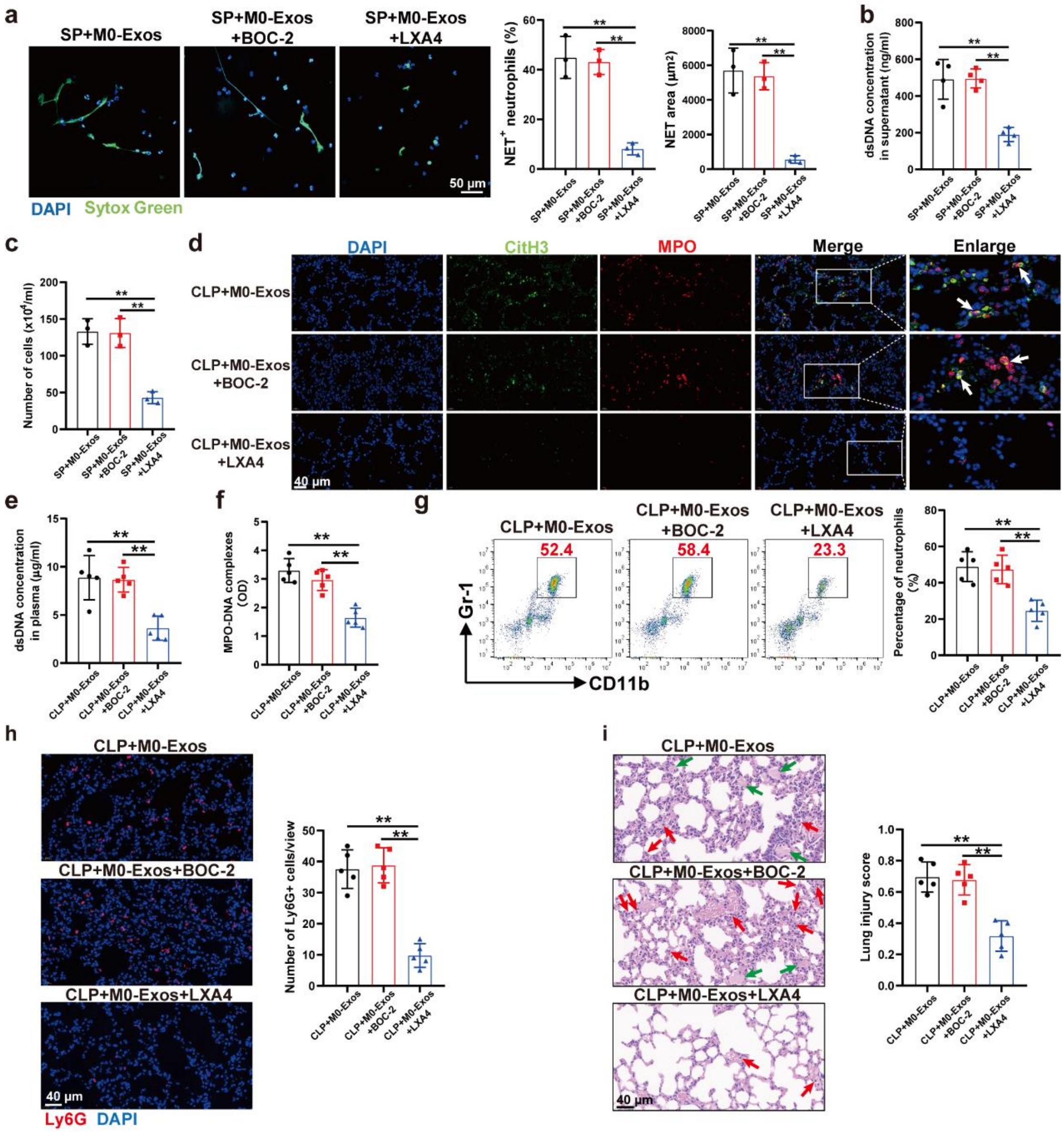

Figure S6 Exogenous addition of LXA4 inhibits PMN recruitment and NET formation during sepsis both *in vitro* and *in vivo*. (**a-c**) PMNs isolated from healthy volunteers were activated by septic plasma (SP), and then cocultured with M0-Exos (100 μg/mL) derived from PBMC-differentiated macrophages for 5 h with or without BOC-2 (10 μM), LXA4 (100 nM), as indicated in the figures. (**a**) Typical images of NET formation using SYTOX Green (green). Scale bar, 50 μm. NET formation was quantified as the percentage of neutrophils forming NETs and NET area per microscopic field. (**b**) Quantification of dsDNA in the supernatant of cultured PMNs using PicoGreen fluorescent dye. (**c**) Transwell analysis of PMN migration capacity. (**d-i**) WT C57BL/6 mice were administered with M0-Exos (300 μg/mouse) derived from mouse Raw264.7 macrophages via intraperitoneal injection 1 hour after CLP. To block LXA4 receptor, mice were treated with 50 μg/kg BOC-2 *i.p.* 30 min before CLP. To exogenous addition of LXA4, mice were treated with 40 μg/kg LXA4 *i.p.* 1 h after CLP. (**d**) Representative images showing the presence of NETs (MPO, red; citrullinated H3, green) in the lung tissues, as indicated by white arrows. Nuclei were counterstained with DAPI (blue). Scale bar, 40 μm. (**e** and **f**) Quantification of dsDNA and circulating NET structures in the plasma of mice using PicoGreen fluorescent dye and MPO-DNA-ELISA, respectively. (**g**) Flow cytometry detection of percentage of systemic circulating PMNs by staining with CD11b and Gr-1. (**h)** Ly6G^{+} cells in the lung tissues were detected by immunofluorescence. Scale bar, 40 μm. (**i)** Evaluation of lung histology by H&E staining (magnifcation×400). Red arrows indicate neutrophils in the alveolar and interstitial space, green arrows indicate proteinaceous debris filling. Scale bar, 40 μm. Lung injury scores were assessed. One-way analysis of variance with Tukey's multiple comparisons test was used for the analysis. Graphs represent means ± standard deviations, n ≥ 3; *P < 0.05, **P < 0.01 compared within two groups.

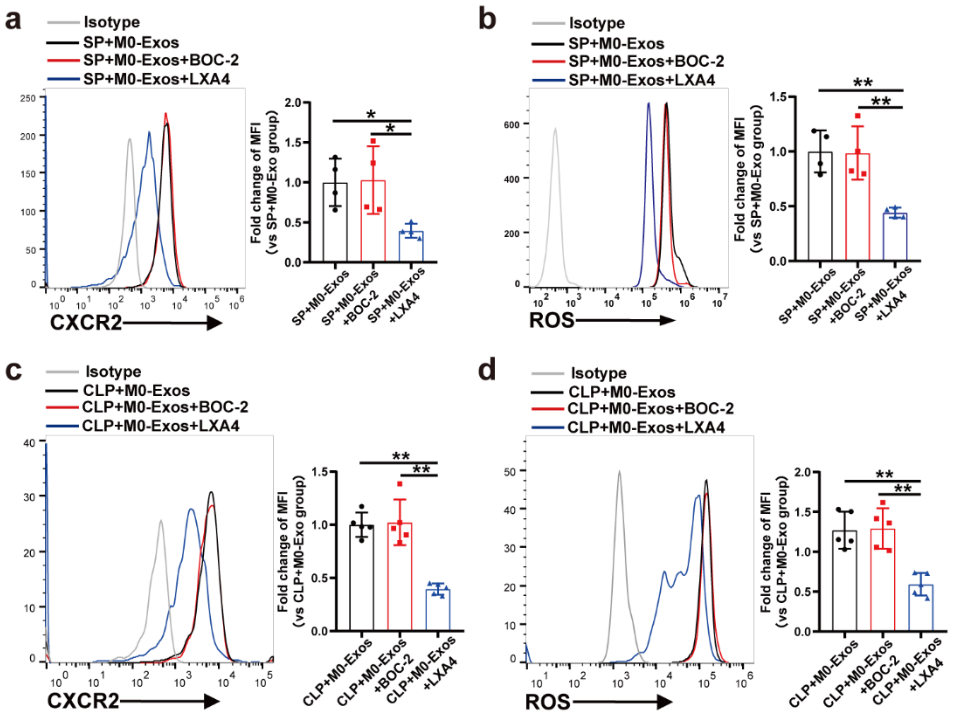

Figure S7 Exogenous addition of LXA4 decreased the expression of CXCR2 and ROS in PMNs during sepsis both *in vitro* and *in vivo*. (**a-b**) PMNs isolated from healthy volunteers were activated by septic plasma (SP), and then cocultured with M0-Exos (100 μg/mL) derived from PBMC-differentiated macrophages for 5 h with or without BOC-2 (10 μM), LXA4 (100 nM), as indicated in the figures. CXCR2 (**a**) and ROS (**b**) expressions in cocultured PMNs were detected by flow cytometry. (**c** and **d**) WT C57BL/6 mice were administered with M0-Exos (300 μg/mouse) derived from mouse Raw264.7 macrophages via intraperitoneal injection 1 hour after CLP. To block LXA4 receptor, mice were treated with 50 μg/kg BOC-2 *i.p.* 30 min before CLP. To exogenous addition of LXA4, mice were treated with 40 μg/kg LXA4 *i.p.* 1 h after CLP. CXCR2 (**c**) and ROS (**d**) expressions in peripheral blood neutrophils were detected by flow cytometry. One-way analysis of variance with Tukey's multiple comparisons test was used for the analysis. Graphs represent means ± standard deviations, n ≥ 3; **P* < 0.05, ***P* < 0.01 compared within two groups.

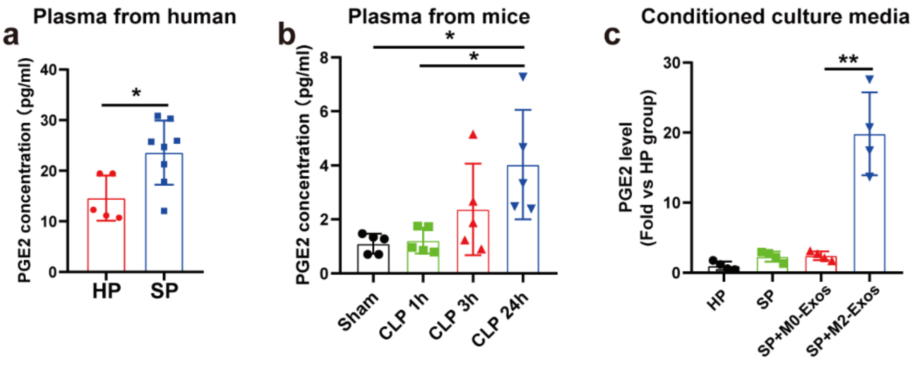

Figure S8 The concentration of PGE2. (**a**) PGE2 concentration in the plasma of healthy volunteers (HP, n = 5) and sepsis patients (SP, n = 8) was measured by ELISA. (**b**) PGE2 concentration in the plasma of sham mice and CLP mice (1 h/3 h/24 h after CLP). (**c**) PMNs isolated from healthy

volunteers were activated upon adding 20% septic plasma (SP) to the culture medium, and 20% plasma from healthy volunteers (HP) was used as negative control. After 1 h, the culture medium was replaced with fresh medium and PMNs were then cocultured with PBS/M0-Exos/M2-Exos (100 μg/mL) derived from PBMC-differentiated macrophages for 5 h. PGE2 level in the supernatant of cocultured PMNs was detected by ELISA. One-way analysis of variance with Tukey's multiple comparisons test (**b** and **c**) or student's t test (**a**) was used for the analysis. Graphs represent means ± standard deviations, n ≥ 3; **P* < 0.05, ***P* < 0.01 compared within two groups.

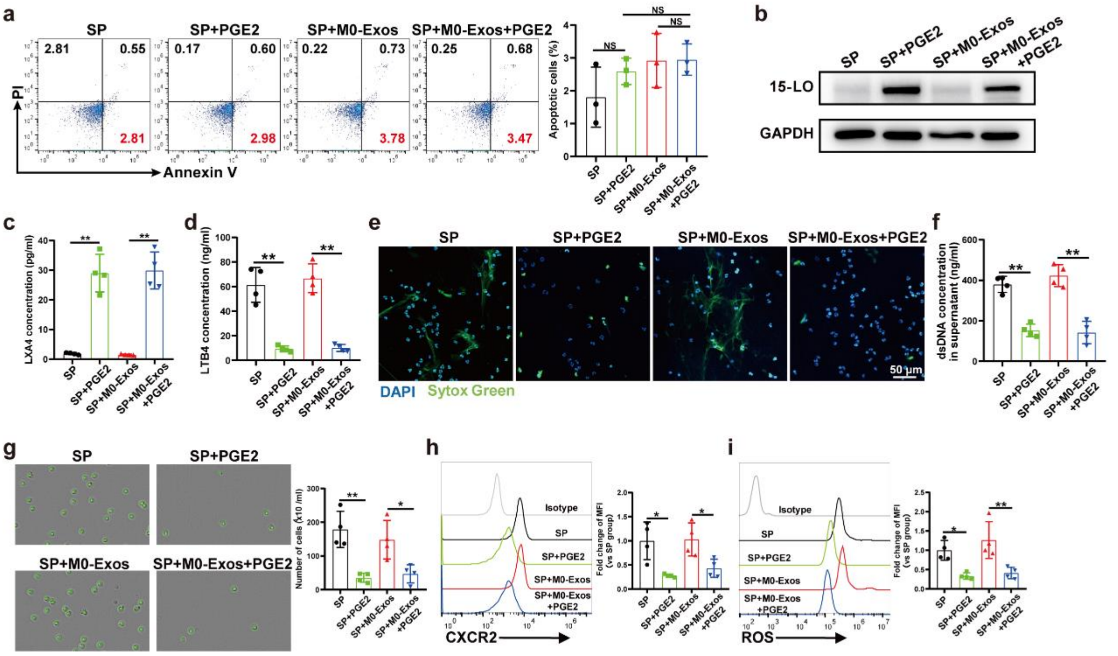

Figure S9 The direct effects of PGE2 on PMNs during sepsis *in vitro*. PMNs isolated from healthy volunteers were activated by septic plasma (SP), and then cocultured with PBS/M0-Exos (100 μg/mL) derived from PBMC-differentiated macrophages for 5 h with or without PGE2 (100 nM), as indicated in the figures. (**a**) Representative flow cytometry plots of Annexin V/PI staining of PMNs, and analysis of Annexin V positive/PI negative-stained cells by apoptotic cells. (**b**) Immunoblot analysis of 15-LO in PMNs. (**c** and **d**) LXA4 and LTB4 concentrations in the supernatant of PMNs were detected by ELISA. (**e**) Typical images of NET formation using SYTOX Green (green). Scale bar, 50 μm. (**f**) Quantification of dsDNA in the supernatant of cultured PMNs using PicoGreen fluorescent dye. (**g**) Transwell analysis of neutrophil chemotaxis towards IL-8. Flow cytometry detection of CXCR2 (**h**) and ROS (**i**) expressions in cocultured PMNs. One-way analysis of variance with Tukey's multiple comparisons test was used for the analysis. Graphs represent means ± standard deviations, n = 3-4; **P* < 0.05, ***P* < 0.01 compared within two groups; NS, not significant.

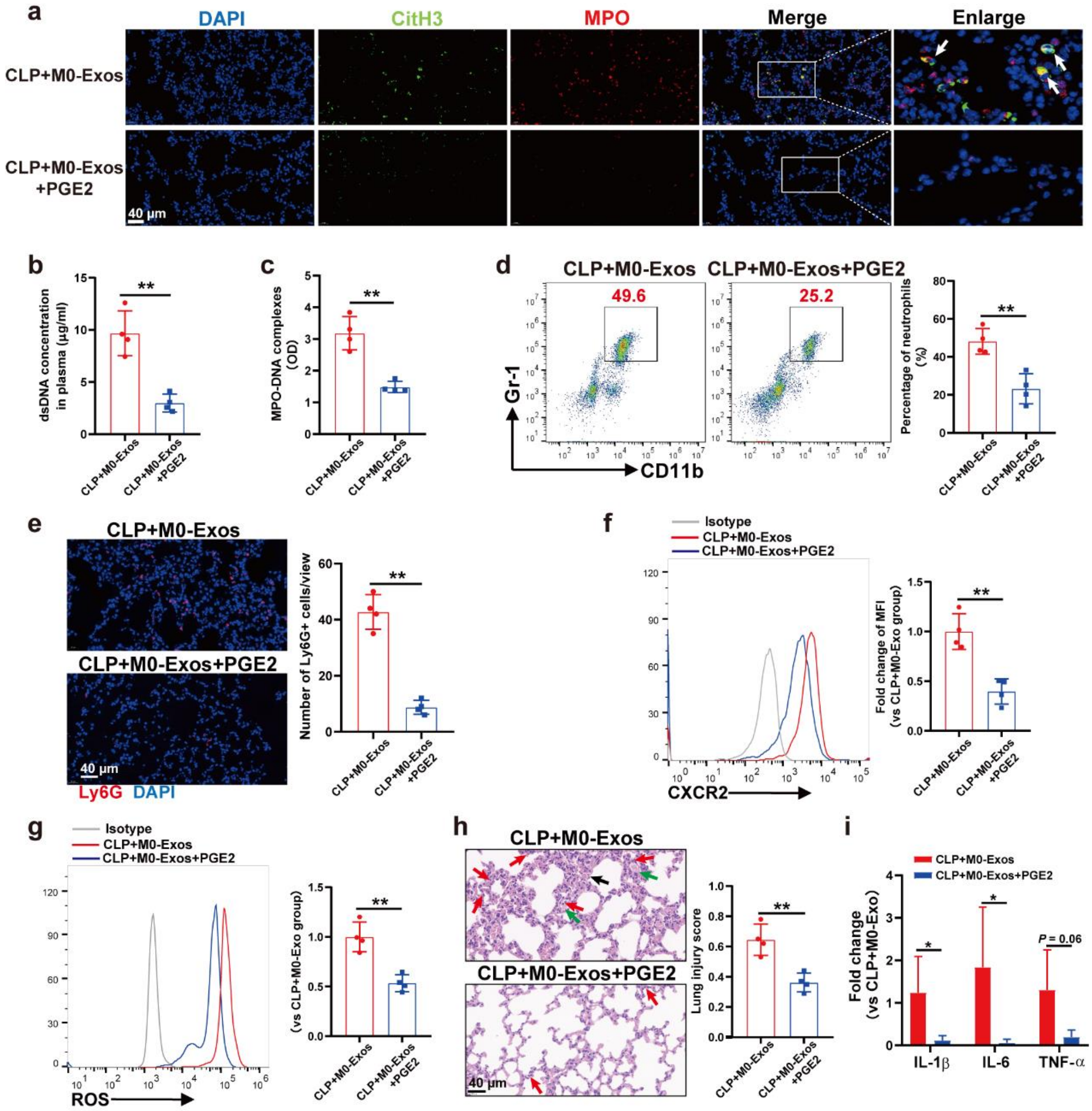

Figure S10 The direct effects of PGE2 on PMNs during sepsis *in vivo*. WT C57BL/6 mice were administered with M0-Exos (300 μg/mouse) derived from mouse Raw264.7 macrophages via intraperitoneal injection 1 hour after CLP. To exogenous addition of PGE2, mice were treated with 2 mg/kg PGE2 *i.p.* 1 h after CLP. (**a**) Representative images showing the presence of NETs (MPO, red; citrullinated H3, green) in the lung tissues, as indicated by white arrows. Nuclei were counterstained with DAPI (blue). Scale bar, 40 μm. (**b** and **c**) Quantification of dsDNA and circulating NET structures in the plasma of mice using PicoGreen fluorescent dye and MPO-DNA-ELISA, respectively. (**d**) Flow cytometry detection of the percentage of systemic circulating neutrophils by staining with CD11b and Gr-1. (**e**) Ly6G$^+$ cells in the lung tissues were detected by immunofluorescence. Scale bar, 40 μm. CXCR2 (**f**) and ROS (**g**) expressions in peripheral blood neutrophils were detected by flow cytometry. (**h)** Evaluation of lung histology by H&E staining (magnifcation×400). Red arrows indicate neutrophils in the alveolar and interstitial space, green arrows indicate proteinaceous debris filling and black arrows indicate thickening of the alveolar walls. Scale bar, 40 μm. Lung injury scores were assessed. (**i)** Detection of inflammatory cytokine mRNA (IL-1β, IL-6, TNF-α) expression in the lung tissues by RT-qPCR. Student's t test was used

for the analysis. Graphs represent means ± standard deviations, n = 4. *P < 0.05, **P < 0.01 compared within two groups.

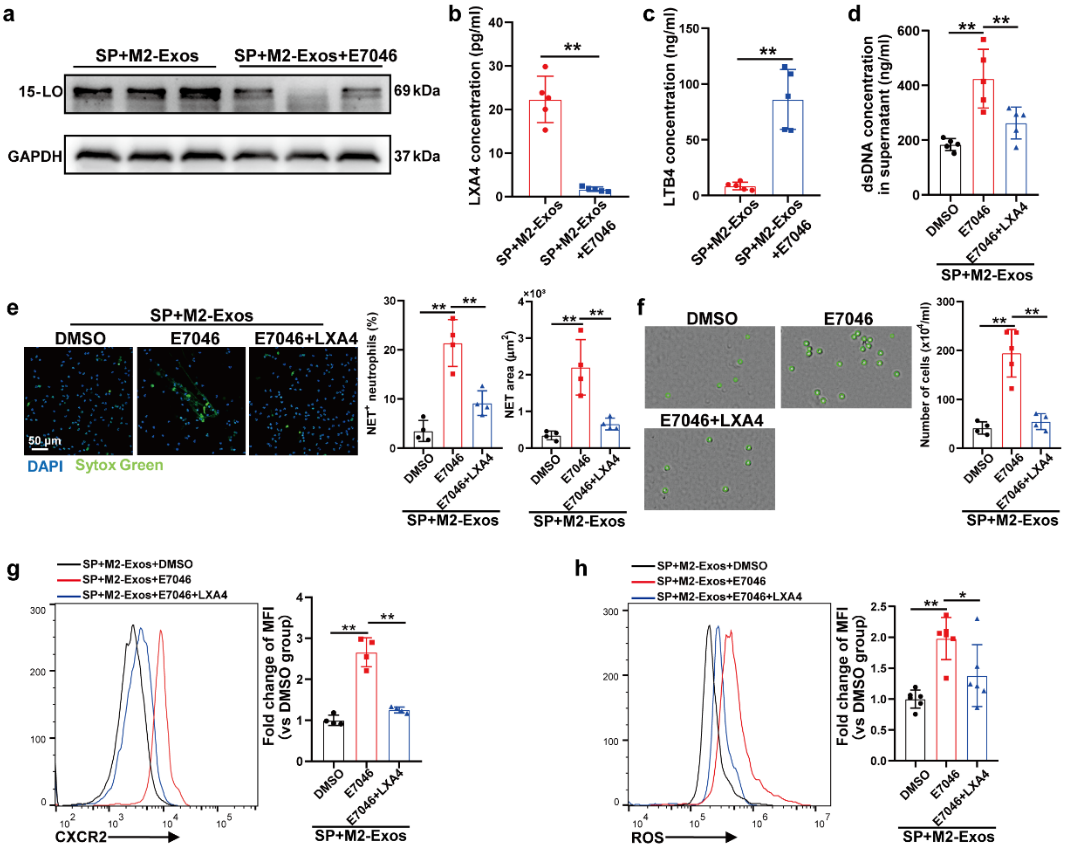

Figure S11 Exosomal PGE2 from M2 macrophage functions on EP4 receptor of PMNs *in vitro*. PMNs isolated from healthy volunteers were activated by septic plasma (SP), and then cocultured with M2-Exos (100 μg/mL) derived from PBMC-differentiated macrophages for 5 h with or without E7046 (1 μM), LXA4 (100 nM), as indicated in the figures. DMSO was used as negative control. (**a**) 15-LO expression in PMNs was detected by Western blot. (**b** and **c**) LXA4 and LTB4 concentrations in the supernatant of PMNs were detected by ELISA. (**d**) Quantification of dsDNA in the supernatant of cultured PMNs using PicoGreen fluorescent dye. (**e**) Typical images of NET formation using SYTOX Green (green). Scale bar, 50 μm. NET formation was quantified as the percentage of neutrophils forming NETs and NET area per microscopic field. (**f**) Transwell analysis of neutrophil chemotaxis towards IL-8. Flow cytometry detection of CXCR2 (**g**) and ROS (**h**) expressions in cocultured PMNs. Student's t test (**b** and **c**) or one-way analysis of variance with Tukey's multiple comparisons test (**d-h**) was used for the analysis. Graphs represent means ± standard deviations, n = 4-5; *P < 0.05, **P < 0.01 compared within two groups.

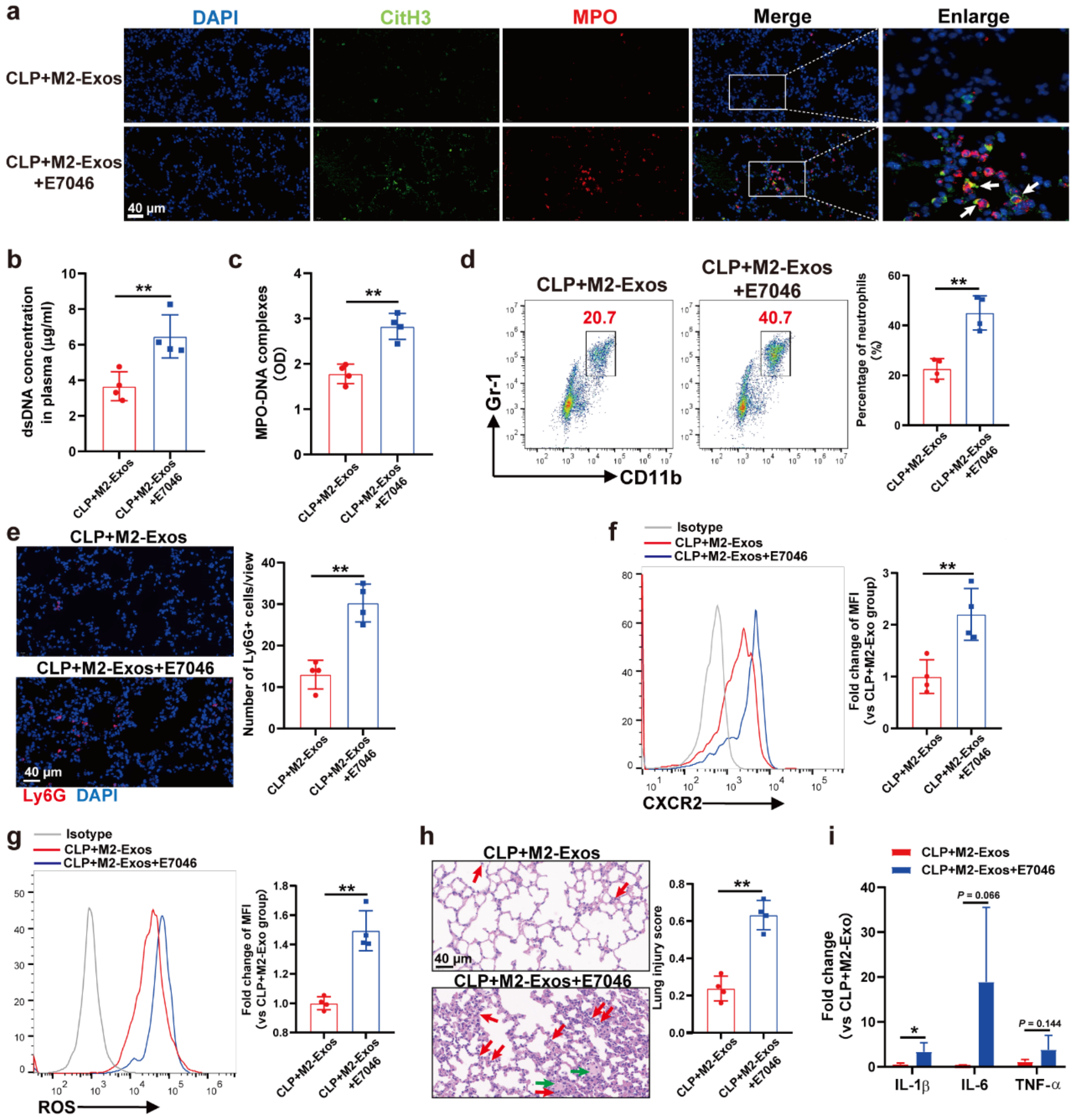

Figure S12 Exosomal PGE2 from M2 macrophage functions on EP4 receptor of PMNs *in vivo*. WT C57BL/6 mice were administered with M2-Exos (300 μg/mouse) derived from mouse Raw264.7 macrophages via intraperitoneal injection 1 hour after CLP. To block EP4 receptor, mice were treated with 10 mg/kg E7046 *i.p.* 30 min before CLP. (**a**) Representative images showing the presence of NETs (MPO, red; citrullinated H3, green) in the lung tissues, as indicated by white arrows. Nuclei were counterstained with DAPI (blue). Scale bar, 40 μm. (**b** and **c**) Quantification of dsDNA and circulating NET structures in the plasma of mice using PicoGreen fluorescent dye and MPO-DNA-ELISA, respectively. (**d**) Flow cytometry detection of the percentage of systemic circulating neutrophils by staining with CD11b and Gr-1. (**e**) Ly6G^{+} cells in the lung tissues were detected by immunofluorescence. Scale bar, 40 μm. CXCR2 (**f**) and ROS (**g**) expressions in peripheral blood neutrophils were detected by flow cytometry. (**h)** Evaluation of lung histology by H&E staining (magnifcation×400). Red arrows indicate neutrophils in the alveolar and interstitial space, green arrows indicate proteinaceous debris filling and black arrows indicate thickening of the alveolar walls. Scale bar, 40 μm. Lung injury scores were assessed. (**i**) Detection of inflammatory

cytokine mRNA (IL-1β, IL-6, TNF-α) expression in the lung tissues by RT-qPCR. Student's t test was used for the analysis. Graphs represent means ± standard deviations, $n = 4$. $^*P < 0.05$, $^{**}P < 0.01$ compared within two groups.
