# Supplementary material for: Exosomal PGE2 from M2 macrophages inhibits neutrophil recruitment and NET formation through lipid mediator class switching in sepsis
Source: J Biomed Sci. 2023 Aug 2;30:62. doi: 10.1186/s12929-023-00957-9 (PMC10394797; doi:10.1186/s12929-023-00957-9)
Supplement: Supplementary file 2 — Additional file 2. Supplementary methods. [file 12929_2023_957_MOESM2_ESM.pdf]

Supplementary Methods

Exosome characterization

The morphology of the exosomes was characterized using transmission electron microscopy. The size distribution of exosomes was assessed by NanoSight tracking analysis. The surface markers of exosomes (CD9, CD63 and TSG101) were identified by western blot, loading with the same amount of exosome protein (60 μg).

Transmission electron microscopy

After isolation of exosomes, the pellets were fixed with 2% glutaraldehyde in 0.1 M phosphate buffer (pH 7.4). The fixed pellets were placed on 100-mesh, carbon-coated, formvar-coated nickel grids treated with poly-L-lysine for 30 min. After washing the samples with several drops of PBS, samples were incubated with drops of buffered 1% glutaraldehyde for 5 min and then washed several times with drops of distilled water. Afterward, samples were negatively stained with drops of Millipore-filtered aqueous 4% uranyl acetate for 5 min. Stain was blotted dry from the grids with filter paper, and samples were allowed to dry. The microscopy images were captured by a JEOL JEM-1400 transmission electron microscope operating at 120 kV.

Western blot analysis

Cells were lysed in ice-cold RIPA Lysis buffer supplemented with protease inhibitor cocktail, and the protein concentration was determined by BCA assay. Cell lysates were then boiled in SDS sample buffer and resolved on 10-12% SDS-PAGE gel. Immunoblots were incubated overnight with primary antibodies against CD9 (#A19027; ABclonal, Wuhan, China), CD63 (#A5271; ABclonal), TSG101 (#A1692; ABclonal), ALOX15 (#A6864; ABclonal), ALOX5 (#A2877; ABclonal), GAPDH (#5174; Cell Signaling Technology). Immunoblots were examined using an ECL detection reagent (#WBULS0500; Millipore Corporation, Billerica, MA, USA).

Fluorescence imaging of Dil-exosomes

Every 10 μg of isolated exosomes were incubated with 1 μl Dil labeling solution (#V22885, Thermo Fisher Scientific) for 30 min at 37℃. Then, the Dil-exosomes were precipitated again using ExoQuick-TC (#EXOTC10A-1; System Biosciences) according to the manufacturer’s protocol. Dil-exosomes were then resuspended in 200 µL PBS buffer and injected into mice (*i.p.*). After 24 h, the lungs were dissected for *ex vivo* evaluation and the biodistribution of Dil-exosomes in the lungs was monitored using IVIS Spectrum In Vivo Imaging System (PerkinElmer, Waltham, MA, USA). Filters allowing excitation at 560 nm and collection of emission at 590 nm were used to obtain ideal images.
